# Biomarker Assessment of Homologous Recombination Deficiency in Epithelial Ovarian Cancer: Association With Progression-Free Survival After Surgery

**DOI:** 10.3389/fmolb.2022.906922

**Published:** 2022-06-13

**Authors:** Huan Yi, Linhong Li, Jimiao Huang, Zhiming Ma, Hongping Li, Jian Chen, Xiangqin Zheng, Jingjing Chen, Haixin He, Jianrong Song

**Affiliations:** ^1^ Department of Gynecology Oncology, Fujian Key Laboratory of Women and Children’s Critical Diseases Research [Fujian Maternity and Child Health Hospital(Fujian Women and Children’s Hospital)], Fujian Maternity and Child Health Hospital College of Clinical Medicine for Obstetrics and Gynecology and Pediatrics, Fujian Medical University, Fuzhou, China; ^2^ Department of Gynecology Oncology, Fujian Provincial Cancer Hospital, Fuzhou, China; ^3^ Research and Development Division, Oriomics Biotech Inc, Hangzhou, China; ^4^ Department of Obstetrics and Gynecology, Fuding General Hospital, Fuding, China

**Keywords:** epithelial ovarian cancer, homologous recombination deficiency, homologous recombination deficiency score, chromosomal instability, postoperative progression-free survival

## Abstract

Identifying BRCA mutations and homologous recombination deficiency (HRD) is the key to choosing patients for poly (ADP-ribose) polymerase inhibitor (PARPi) therapy. At present, a large amount of research focuses on the application of HRD detection in ovarian cancer. However, few studies have discussed the relationship between HRD detection and postoperative survival in patients with epithelial ovarian cancer (EOC). This study included 38 consecutive patients with EOC who underwent cytoreduction surgery. Owing to tissue availability, only 29 patients underwent molecular profiling and survival analysis. Overall, 21 (72.4%) tumors had HRD scores of ≥42. Mutations in BRCA were observed in 5/29 (17.2%) patients. In this cohort, an HRD score of ≥42 was more common in serous ovarian tumors. We found no statistically significant association between homologous recombination repair (HRR) genes and HRD scores except for tumor protein P53 (TP53) mutation. We also found a strong positive association between HRD scores and chromosomal instability (CIN). In the survival analysis, an HRD score of >23 was correlated with better postoperative progression-free survival (pPFS). With increased depth of research, an appropriate HRD score threshold may serve as a prognostic tool and should be assessed in future studies to predict the clinical value of PARPi.

## Introduction

Ovarian cancer is the third most frequent gynecologic cancer and the leading cause of gynecologic cancer death in China (Association, 2021; Sung et al., 2021). Due to the lack of effective early screening methods, most patients are diagnosed at a late stage. The 5-years survival rate is lower than 40% in China (Jiang et al., 2018). The World Health Organization (WHO) classifies ovarian cancers into those involving epithelial cells, germ cells, and mesenchyme (Kaku et al., 2003). Epithelial ovarian cancer (EOC) is the major type of ovarian malignancy, and its histologic classifications is as follows: high-grade serous (HGS) (up to 70%), low-grade serous, endometrioid, clear cell, and mucinous (Ledermann et al., 2016). The primary standard treatment approaches for EOC, such as debulking surgery and adjuvant platinum chemotherapy, can be used to achieve good initial response rates, but most ovarian cancer patients will eventually relapse (Deraco et al., 2012; Hiss, 2012; Zheng and Gao, 2012).

The homologous recombination (HR) pathway is an important DNA damage response mechanism that safeguards genome stability by repairing double-strand DNA breaks with high fidelity. Among the numerous albuminoidal factors of the HR pathway, BRCA proteins are essential for the complete HR repair response (Wang and Lindahl, 2016; Pastink et al., 2001; del Rivero and Kohn, 2017). Germline or somatic BRCA mutations and BRCA gene promoter methylation are the main causes of homologous recombination deficiency (HRD), but other genetic abnormalities in the homologous recombination repair (HRR) pathway may also contribute to HRD (Xu et al., 2010; Pilié et al., 2019; Kalachand et al., 2020). Furthermore, homologous recombination proficiency (HRP) can be restored by reverse mutation of various HRR pathway genes, such as *BRCA1/2*, *PALB2*, and *RAD51C/D*, indicating that HRD status is a complex and dynamic phenotype (Norquist et al., 2011; Goodall et al., 2017; Kondrashova et al., 2017; Weigelt et al., 2017; Lin et al., 2019). Due to the abnormal function of genes in the HR pathway, cells with HRD are more sensitive to platinum drugs and poly (ADP- ribose) polymerase inhibitors (PARPi) (Telli et al., 2016; Stronach et al., 2018; Stover et al., 2020). In recent years, multiple clinical studies have shown that PARPi treatment produces better progression-free survival (PFS) in patients with *BRCA1/2* mutation (Mirza et al., 2016; Coleman et al., 2017; Pujade-Lauraine et al., 2017). Expanding the use of PARPi by identifying other HRD biomarkers is increasingly the focus of research. Currently, HRD tests can be divided into three categories: 1) HRR pathway-related genes, 2) genomic “scars” or signatures of mutations that represent patterns of genome instability and 3) functional assays that provide a real-time read out of HRD or HRP (Miller et al., 2020; Chiang et al., 2021).

An HRD score ≥42 is correlated with longer survival in EOC patients cured with platinum drugs or PARPi (Stronach et al., 2018; Chen et al., 2021). However, it is uncertain whether HRD status is associated with postoperative PFS (pPFS) in EOC. Therefore, we investigated the relationship between HRD status and prognosis in postoperative EOC patients, and we found that postoperative EOC can be divided into high and low-risk groups based on HRD status.

## Methods

### Study Populations

A consecutive series of 38 EOC patients who underwent surgical treatment from the Fujian Maternal and Child Health Hospital (from July 2017, to July 2021) were prospectively enrolled for HRD analyses. We included biopsy-proven epithelial ovarian cancer patients with sufficient tissue sample collection (five to ten 15 micron formalin-fixed paraffin embedded (FFPE) tissue sections) for molecular testing. Patients without sufficient tumor specimens and clinical information were excluded from this study. Chemotherapy regimens for patients receiving neoadjuvant chemotherapy (NACT) or primary cytoreductive surgery (PCS) included cisplatin, carboplatin, Nida platinum and paclitaxel. The study was permitted by the local Ethics Committees of the Fujian Maternal and Child Health Hospital, and informed consent was acquired from each patient. Further details of the enrolled patients are shown in Table 1.

**TABLE 1:** Demographic and clinical characteristics (n = 29).

Clinicopathologic characteristic	N	%
Age		
Median (range)	52 (34–70)	
Stage		
I	3	10.4
II	4	13.7
III	19	65.5
IV	3	10.4
Histology		
High-grade serous	22	75.8
Endometrioid	3	10.4
Clear cell	2	6.9
Adenocarcinoma NOS^a^	2	6.9

a NOS, not otherwise specified.

### Clinical Data Collection

Patients were followed up from July 2013 to December 2021 every 6 months. Clinical information was acquired from the internal database of the hospital. PFS was assessed based on Response Evaluation Criteria in Solid Tumours (RECIST) version 1.1 criteria, measured as the time from primary surgery to disease progression or death as a result of any cause, whichever was earlier, and ultrasonography, computed tomography (CT), and magnetic resonance imaging (MRI) were performed at baseline and after six to eight cycles of chemotherapy (Bogaerts et al., 2009).

### Capture-Based Targeted DNA Sequencing

DNA from FFPE specimens was purified with the TIANamp FFPE DNA Kit (DP331, TIANGEN Biotechnology, China) according to the manufacturer’s instructions. The extracted DNA specimens were quantified with a Qubit dsDNA HS Assay Kit (Invitrogen) and cleaved into 150–200-bp fragments with Covaris M220 (Covaris, Brighton, United Kingdom). Qsep100 (Bioptic, Taiwan, China) was used for fragment quality control. The damaged DNA was repaired by a VAHTS^®^ DNA Damage Repair Kit (Vazyme, Nanjing, China). A DNA fragment library was created by a nano DNA library preparation kit (Nanodigmbio, Nanjing, China), and then PCR amplification and purification were performed. Afterwards, the library was captured by a x-Gen^®^ Hybridization and Wash Kit (IDT). The designed HRD panel contained 112 genes and 32,501 single nucleotide polymorphisms (HRDcare^®^, Oriomics Biotech Inc, Zhejiang, China). NadPrep NanoBlocker (Nanodigmbio, Nanjing, China) was then used to block the barcoded adapters. A Qubit 3.0 fluorimeter (Invitrogen) and Qsep100 were used for the final library quality control. Sequencing was performed on an Illumina NextSeq 550 Dx platform, and the average coverage depth of the captured area was 150×.

### Analysis of Sequencing Data

Fastp 0.20.0 software was used to evaluate the quality of the raw sequencing data (Chen et al., 2018). The sequencing data were aligned to HG19 utilizing Burrows–Wheeler Aligner v.0.7.17. AMtools v.1.7 was utilized to transform SAM files to BAM files and to dispose the BAM files by chromosomal coordinates (Li et al., 2009). Copy number variants (CNVs) and single nucleotide variation (SNV) identification was performed using facets v.0.16.0 and VarDict v.1.8.2. Mutations were then filtered according to the following criteria (Association, 2021): variant allele frequency (VAF) greater than 1% with at least three supporting reads; and (Sung et al., 2021) ExAC, 1,000 Genomes or dbSNP databases of <0.05. The remaining variants were annotated with ANNOVAR(Ng and Henikoff, 2003). The HRD score, including assessments of loss of heterozygosity, telomeric allelic imbalance, and large-scale state transition, was calculated utilizing the scarHRD software, as previously described (Telli et al., 2016; Sztupinszki et al., 2018; (Timms et al., 2014).

### Analysis of the Differentially Expressed Genes and Functional Enrichment Analyses

*The* Cancer Genome Atlas (*TCGA*) *EOC data set was used to examine the differences in mRNA levels between the high (≥ 42) and low (< 42) HRD score groups using a cutoff padj* value of *< 0.05 and a |*log*2FoldChange|* value of > *1. DESeq2 (version 1.24.0) was used to acquire differentially expressed genes (DEGs). Gene Ontology (GO) and Kyoto Encyclopedia of Genes and Genomes (KEGG) pathway enrichment analyses of the DEGs were conducted by clusterProfiler (version 3.12.0).*

### Statistical Methods

All statistical analyses were performed using the R software package (version 3.6.1). Kaplan-Meier analyses were used to evaluate the relationship between gene mutation status and PFS based on the survival package (version 3.1–11). The survminer package (version 0.4.6) was used to determine the optimal cutoff point for the HRD score. All *p* values were two-sided unless otherwise noted, and values <0.05 were considered statistically significant.

## Results

### Clinical Characteristics of the Patients

Thirty-eight patients with EOC were analyzed for HRD score and *BRCA1* and *BRCA2* mutation status. All patients underwent NACT or PCS, and some patients subsequently received interval cytoreductive surgery. The research predated PARPi approvals for first-line maintenance treatment; therefore, few patients chose this therapy. The median age of the EOC patients was 52 years (range 34–70 years). Most patients had stage III and IV disease (75.9%) and serous histology (75.8%). The median PFS after the primary surgery was 13 months (range 2–79 months). All patients had a Karnofsky performance scale (KPS) score of 90–100 and a Zubrod performance status (ZPS) score of 0–1 at baseline.

### Characterization of HRD

**FIGURE 1:**
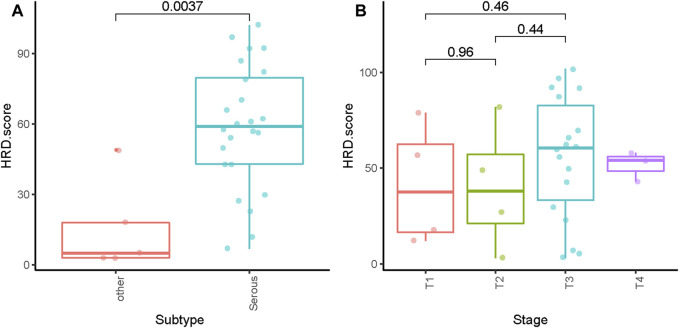
Distribution of HRD scores with different histologies **(A)** and tumor stages **(B)**. ** indicates *p* < 0.01; ns indicates no significant difference.

The HRD score analysis was successful in 29/38 samples (76.3%). Nine patients were excluded because of their low tumor DNA content. Patient demographics and clinical data are shown in Table 1. For the 29 tumor tissue samples that met the experimental requirements, we calculated the HRD score for each sample, referring to the three chromosomal abnormalities previously approved by the FDA in Myriad’s myChoice HRD, which included LOH, TAI, and LST. HRD positivity was determined by either a test value of ≥42 or the presence of a *BRCA* pathogenic or likely pathogenic mutation. Overall, 21 (72.4%) tumor samples had HRD scores of ≥42, which is similar to a previous report (Chen et al., 2021). The proportion of HRD scores of ≥42 in our study was higher than that reported in Western countries (Patel et al., 2018). Mutations in BRCA were observed in 5/29 (17.2%) patients. An HRD score of ≥42 was more common in serous ovarian tumors in our cohort (*p* = 0.0037; Figure 1A). Patients with advanced ovarian tumors had a higher proportion of HRD scores of ≥42, although this result was not statistically significant (Figure 1B).

### HRR Gene Mutation and Copy Number Variation

**FIGURE 2:**
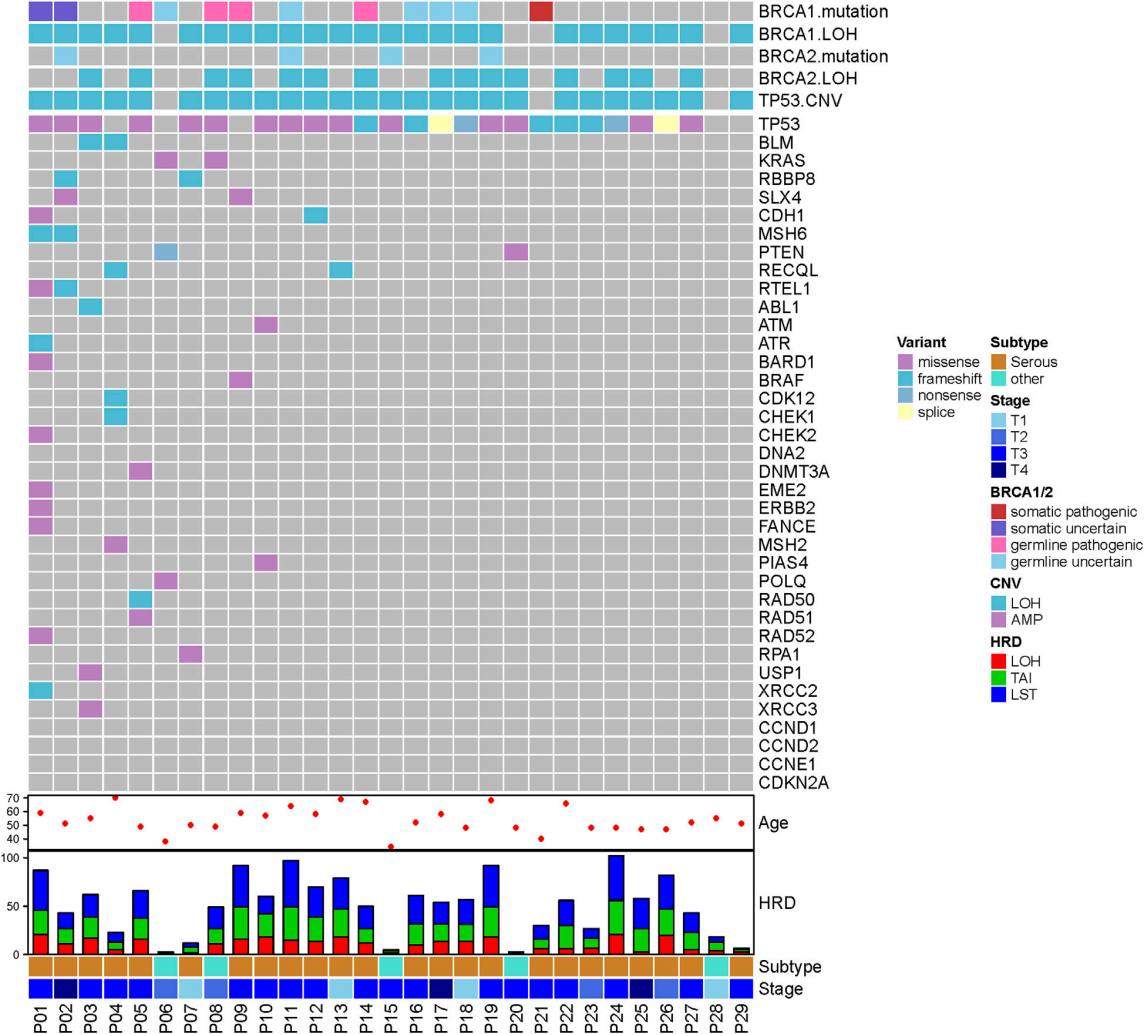
HRR gene mutation landscape and HRD scores.

**FIGURE 3:**
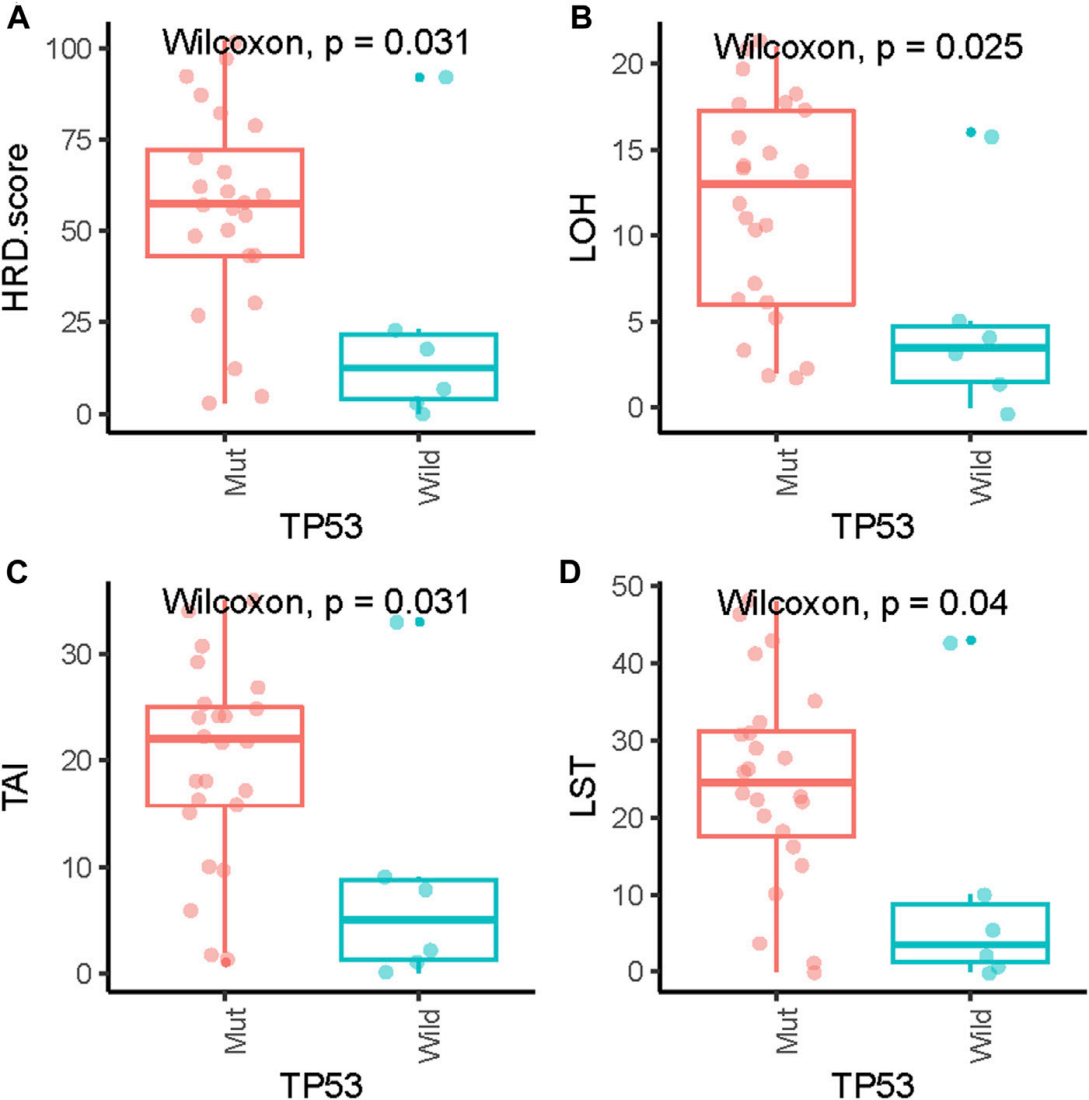
Association of HRD scores **(A)**, loss of heterozygosity (LOH) **(B)**, telomeric allelic imbalance (TAI) **(C)** and large-scale state transition (LST) **(D)** with TP53 gene mutations.

Pathogenic mutations in BRCA were detected in five (5/29, 17.2%) samples and included four germline mutations and one somatic mutation (Table 2). We then evaluated the association between the HRD score and mutations in all HRR pathway genes, not just *BRCA* mutations. These ovarian tumors had universal Tumor Protein P53 (TP53) mutations and many other genetic mutations, most of which were HRR pathway gene mutations (Figure 2). We found no statistically significant associations between the whole mutation statuses of the genes of the HRR pathway and HRD scores. Nevertheless, individual analyses of these HRR mutations showed that TP53 mutation was associated with high HRD scores (Figure 3, *p* = 0.031), which has also been reported in colorectal and prostate cancer ((Smeby et al., 2020; Lotan et al., 2021).

**TABLE 2:** HRD testing characteristics in patients.

Sample	LOH	TAI	LST	HRD Score	BRCA1/2 Mutation
P24	21	35	46	102	NA^a^
P11	15	34	48	97	germline uncertain
P09	18	31	43	92	NA
P19	16	33	43	92	germline pathogenic
P01	21	25	41	87	somatic uncertain
P26	20	27	35	82	NA
P13	18	29	32	79	NA
P12	14	25	31	70	NA
P05	16	22	28	66	germline pathogenic
P03	17	22	23	62	NA
P16	10	22	29	61	germline uncertain
P10	18	24	18	60	NA
P25	3	24	31	58	NA
P18	14	17	26	57	germline uncertain
P22	6	24	26	56	NA
P17	14	18	22	54	germline uncertain
P14	12	15	23	50	germline pathogenic
P08	11	16	22	49	germline pathogenic
P02	5	18	20	43	NA
P27	11	16	16	43	somatic uncertain
P21	6	10	14	30	somatic pathogenic
P23	7	10	10	27	NA
P04	5	8	10	23	NA
P28	4	9	5	18	NA
P07	2	6	4	12	NA
P29	3	2	2	7	NA
P15	2	2	1	5	NA
P06	1	1	1	3	germline uncertain
P20	2	1	0	3	NA

a NA, not applicable.

Next, we analyzed the CNVs of HRR-related genes and identified the relationship between CNV and HRD scores. The higher the HRD score was, the more abnormal the copy number. (Supplementary Figures S1–S29). We also found a significant correlation between HRD scores and chromosomal instability (CIN) (Figure 4, *p* = 4.7 e-09, R = 0.86).

**FIGURE 4:**
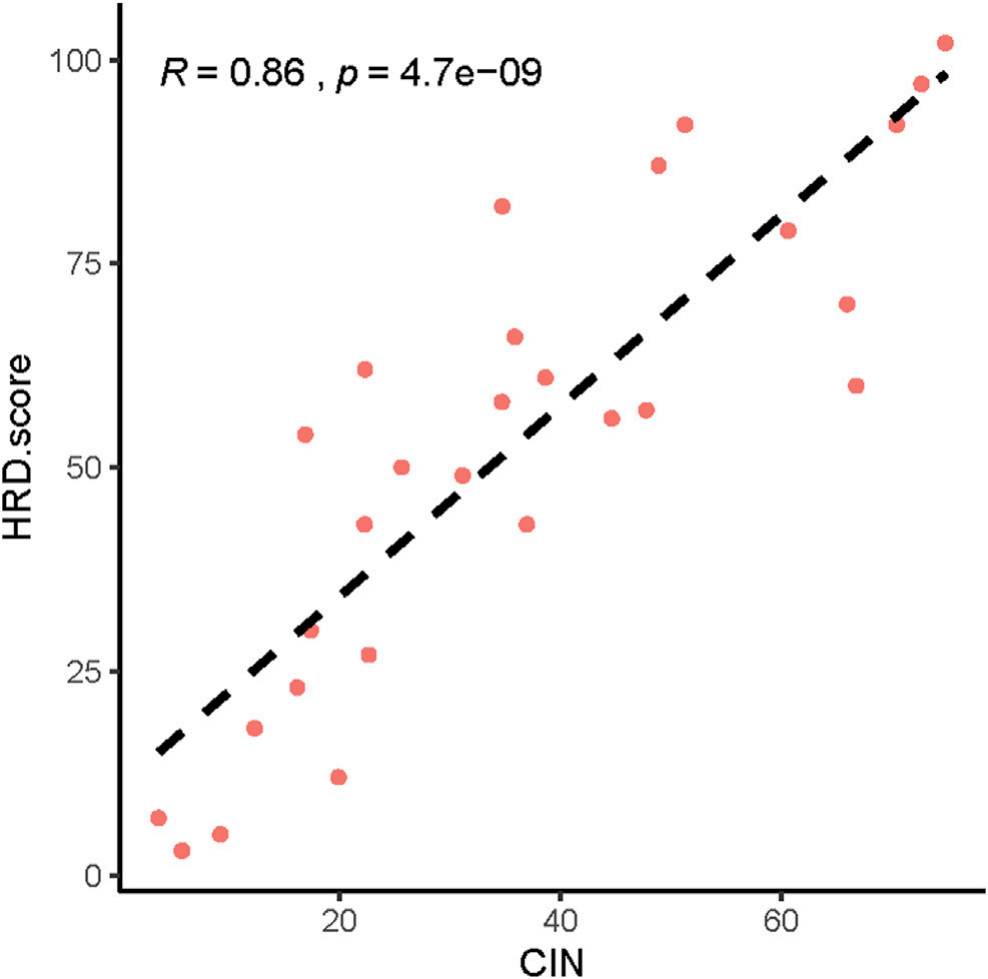
Relationship between HRD scores and chromosomal instability (CIN).

### Survival

**FIGURE 5:**
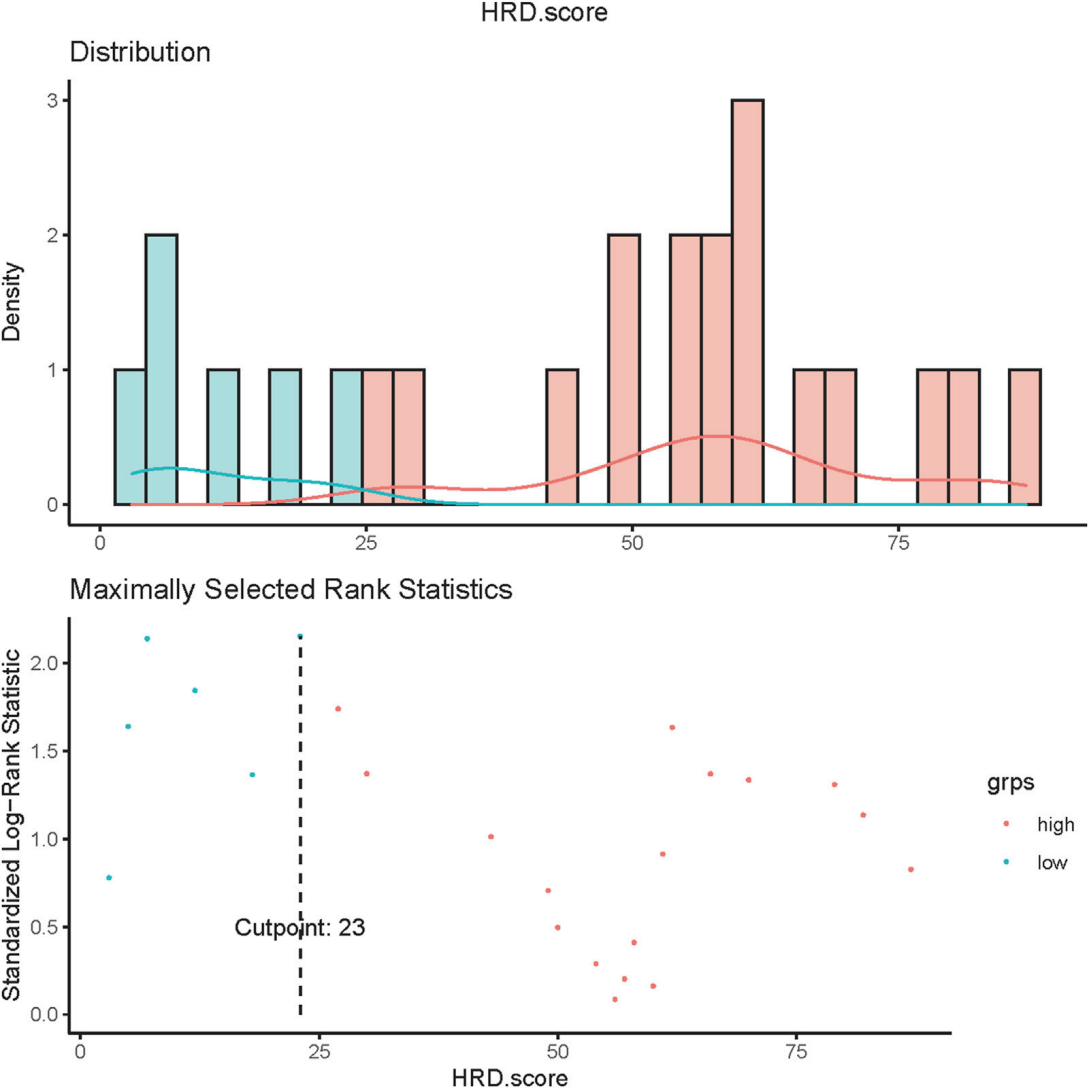
HRD score values in ovarian cancer. Values of HRD scores above 23 (dashed line) were regarded as HRD-high, and values below 23 were regarded as HRD-low.

Of all patients who completed the HRD score assessment (n = 29), 22 patients had an HRD score of>23. Among the 24 patients without somatic or germline *BRCA* mutations who achieved HRD score detection, 17 had an HRD score greater than 23 (Figure 5).

The result of a postoperative progression-free survival (pPFS) univariate analysis utilizing different HRD score thresholds is shown in Table 3. The median pPFS was 13 months (range 2–79 months). Although patients with an HRD score of ≥42 had improved pPFS, there were no significant differences between groups. Notably, we found that an HRD score of >23 was statistically correlated with better pPFS (HR 0.21, 95% CI 0.048–0.97; *p* = 0.046) (Table 3). Multivariate analysis had similar results. An HRD score of >23 (HR 0.019, 95% CI 0.00095–0.36; *p* = 0.008) was significantly associated with improved pPFS when adjusting for histology and TP53 mutation (Table 4).

**TABLE 3:** Postoperative progression-free survival in patients.

Characteristic	N	Hazard Ratio (95% CI)	P
HRD score			
<42	9	Ref	
≥42	20	0.36 (0.08–1.7)	0.19
HRD score			
≤23	7	Ref	
>23	22	0.21 (0.048–0.97)	0.046

**TABLE 4:** Multivariate analysis for postoperative progression-free survival.

Characteristic	N	Hazard ratio (95% CI)	P
HRD score			
≤23	7	Ref	
>23	22	0.019 (0.00095–0.36)	0.008
TP53			
Mut	24	Ref	
Wild	5	0.12 (0.0089–1.6)	0.11
Histology			
Other	5	Ref	
Serous	23	4.00 (0.24–66.56)	0.33
Tumor stage			
I + II	7		
III + IV	19	NA^a^	NA

a NA, not applicable.

**FIGURE 6:**
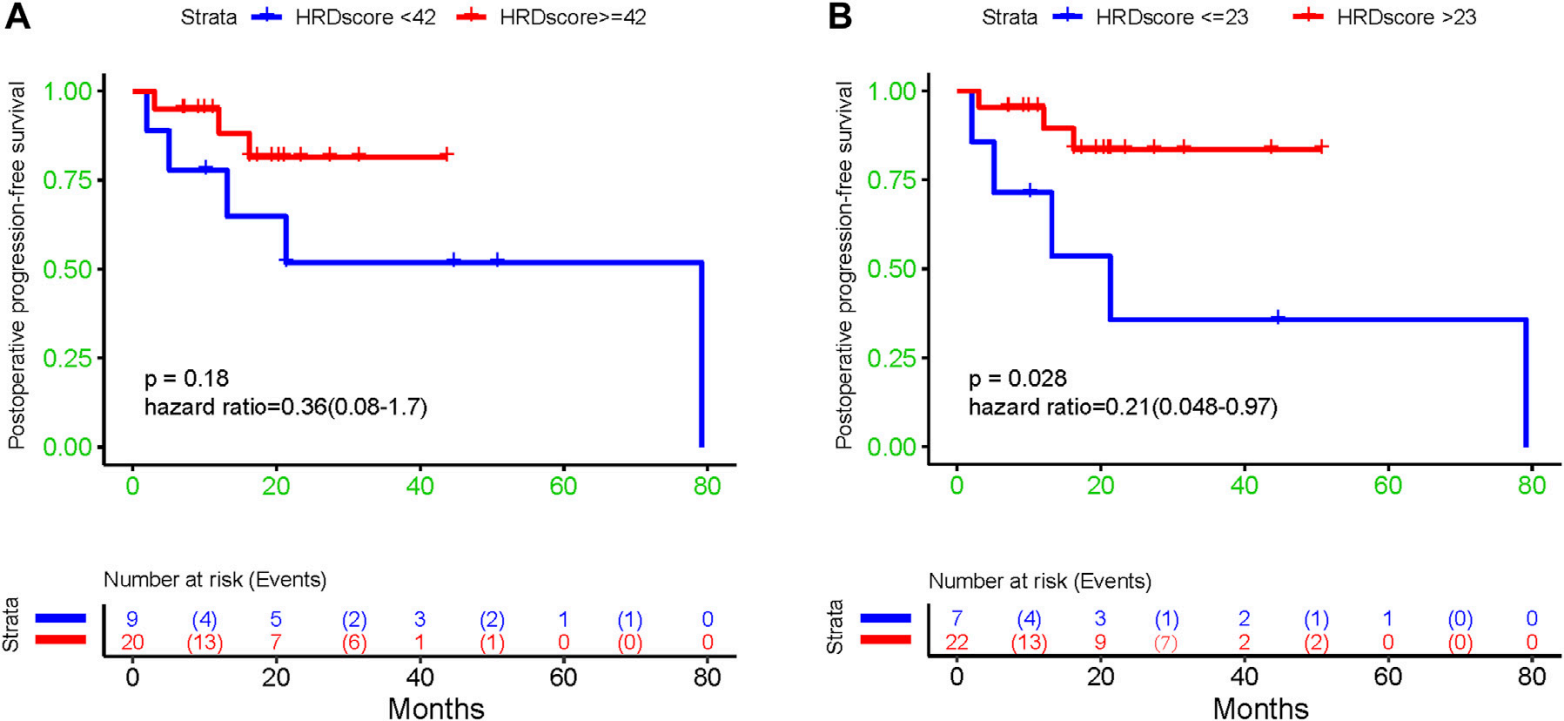
The association between diverse HRD score thresholds and postoperative progression-free survival: **(A)** 42; **(B)** 23.

**FIGURE 7:**
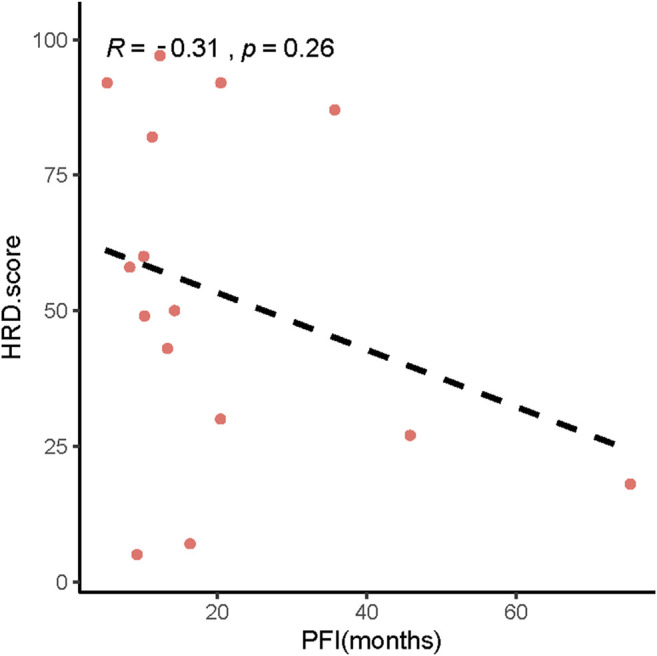
Relationship between HRD scores and platinum-free interval (PFI).

In addition, our results showed that patients with low HRD scores had significantly worse pPFS than those with high HRD scores, which was statistically significant by adjusting the HRD score threshold to 23 (*p* = 0.18, Figure 6A, *p* = 0.028, Figure 6B). In relapsed patients, a higher HRD score was associated with a shorter platinum-free interval (PFI) (Figure 7).

### Functional Enrichment Analysis of DEGs

**FIGURE 8:**
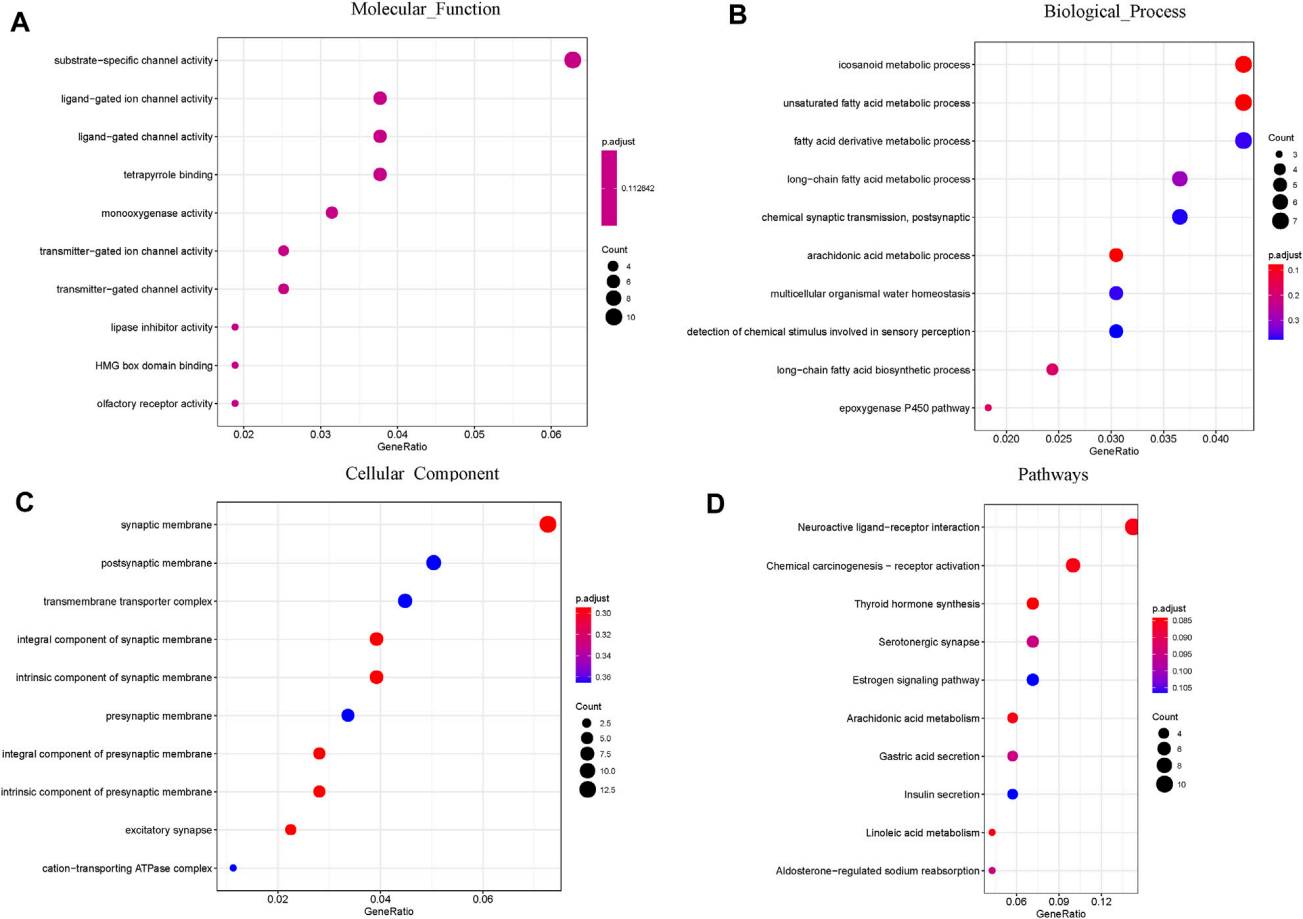
GO and pathway enrichment analysis of the DEGs: **(A)** Molecular Function; **(B)** Biological Process; **(C)** Cellular Component. **(D)** KEGG pathway enrichment analysis of the DEGs.

A total of 474 DEGs were identified, 252 of which were upregulated and 222 of which were downregulated. To further explore the association of the HRD score and survival, GO and KEGG enrichment analyses were conducted. GO enrichment analysis showed that the DEGs between the high and low HRD score groups were remarkably enriched for substrate-specific channel activity, ligand-gated ion channel activity (molecular function [MF]), icosanoid metabolic process, unsaturated fatty acid metabolic process (biological process [BP]), and synaptic membrane and postsynaptic membrane components (cellular component [CC]). KEGG pathway analysis revealed that the DEGs were dominant in neuroactive ligand-receptor interaction and chemical carcinogenesis-receptor activation (Figure 8).

## Discussion

By enabling an assessment of genomic instability and *BRCA* mutations, HRD scores are biomarkers of HR pathway defects, and former studies have shown that these biomarkers predict beneficial responses to DNA-damaging factors in certain tumor types (Telli et al., 2016; Pujade-Lauraine et al., 2017; Mirza et al., 2016). The frontline therapy that is standard for advanced ovarian cancer patients is paclitaxel/carboplatin combined treatment, and the FDA has already approved PARPi for frontline maintenance therapy. With the increasing clinical demand and interest in the assessment of tumor molecular features to treat EOC, we tried to determine the characteristics of HRD biomarkers and their correlation with survival outcomes.

First, the quality of the clinical samples is very important for HRD score detection. In a previous study (Miller et al., 2020), (How et al., 2021), approximately 80%–90% of the samples were completely tested for HRD. Failed detection was frequently due to the poor quality of tissue samples. The percentage of completed testing was lower (29/38, 76.3%) in this study. This result may be due to our relatively poor sample quality. At the same time, we found that high-grade serous ovarian cancer had a higher HRD score than those of other histological ovarian cancers. It has been reported that more than 45% of patients with HGS ovarian cancer may have HRD abnormalities, and the clinical and molecular characteristics of HGS ovarian cancer are associated with genome instability (Moschetta et al., 2016; Hodgson et al., 2018). Our study revealed that the Chinese population had a higher proportion of HRD scores of ≥42 than those of other populations. We suspected that this finding may be due to differences in ethnicity and the different subtypes of ovarian cancer in the study cohort. Furthermore, TP53 gene mutations have been detected in more than 80% of patients. An unexpected finding of our study was the positive association between the TP53 mutation status and the HRD score. RAD51 may be the connection between HRD and wild-type TP53 in EOC. RAD51 plays an important role in homologous recombination by interactingwith DNA, mediating homologous pairing and strand exchange and assisting in double-strand break repair (Arias ‐ Lopez et al., 2006). TP53 downregulates the expression of the crucial HR proteins, including RAD51, thereby inhibiting inapposite DNA repair (Bindra et al., 2004). Nevertheless, prevalent variations in TP53 in tumors lead to overexpression of RAD51 and increased resistance to DNA damage. This finding is consistent with our conclusion, which may interpret the high HRD score in the previously mentioned patients with TP53 variations.

Finally, we analyzed the relationship between the HRD score and PFS after surgery. Some researchers have found that adjusting the HRD threshold may increase the accuracy and the predictive ability of the score for assessing beneficial responses to PARPi or platinum drugs (Stronach et al., 2018; How et al., 2021; Li et al., 2020). In univariate analysis, HRD score thresholds of 42 and 23 were both correlated with better pPFS in EOC patients. Nevertheless, only the result obtained from patients with an HRD score of 23 approached statistical significance (*p* = 0.046). Multivariate analysis showed the same results (*p* = 0.008). Our exploratory research of diverse HRD score thresholds in postoperative EOC tumors showed that HRD status was significantly correlated with pPFS (*p* = 0.028) upon altering the threshold from 42 to 23. This finding indicated that EOC patients with low HRD scores had significantly worse pPFS than those with high HRD scores. Interestingly, we found that the HRD score was negatively correlated with the PFI in relapsed patients. Furthermore, we analyzed the DEGs between EOC patients with high and low HRD scores in TCGA and found that these genes were mainly enriched in GO analysis pathways related to ion channel activity and unsaturated fatty acid metabolic processes. These particular gene expression signatures may be responsible for the positive correlation between the HRD score and the postoperative survival in EOC patients. By performing HRD detection in tissue samples from patients with postoperative EOC, we effectively assessed the postoperative survival of patients. Patients with low HRD scores might have an earlier postoperative recurrence; therefore, postoperative monitoring should be emphasized.

Our study has several limitations. First, our study was conducted with a small specimen size and short follow-up time. However, patient sample collection and follow-up are ongoing. Soon, we will conduct a larger cohort study with a longer follow-up time to fully demonstrate the practical value of HRD detection in Chinese ovarian cancer patients. Second, the adjustment of threshold from 42 to 23 might increase the false positive rate of PARPi-sensitive patients, which may lead to over-treatment and medical resources waste. Finally, further research is required to evaluate the forecasting power of these two thresholds for PARPi treatment benefits.

## Conclusion

In summary, EOC therapy is becoming increasingly personalized, with the increasing knowledge of the molecular characteristics guiding therapy stratification. The HRD scoring test offers a worthy adjunct to gathering evidence of HRD that may have been missed by only detecting somatic BRCA mutation. An HRD score threshold of 23 strongly correlated with an improvement in pPFS unlike the current threshold of 42. This new threshold may help better predict postoperative patient survival.

## Data Availability

The data supporting the results of this research can be obtained from the corresponding authors upon reasonable request.
