# Supplementary figures and images for: Biomarker Assessment of Homologous Recombination Deficiency in Epithelial Ovarian Cancer: Association With Progression-Free Survival After Surgery
Source: Front Mol Biosci. 2022 Jun 13;9:906922. doi: 10.3389/fmolb.2022.906922 (PMC9234295; doi:10.3389/fmolb.2022.906922)

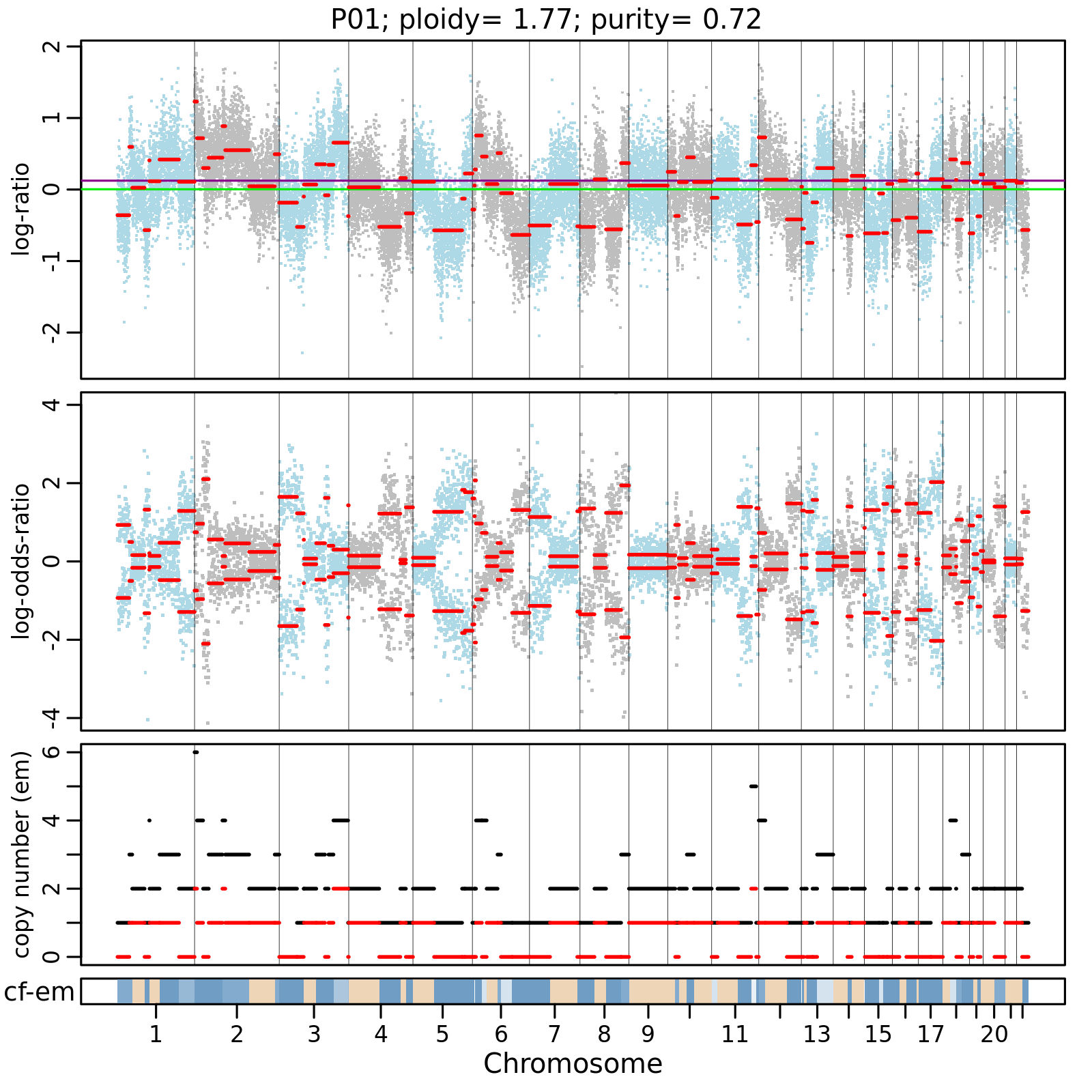

Supplement: Supplementary file 1 [file DataSheet1.ZIP › CNV_plot/P01.cnv.png]

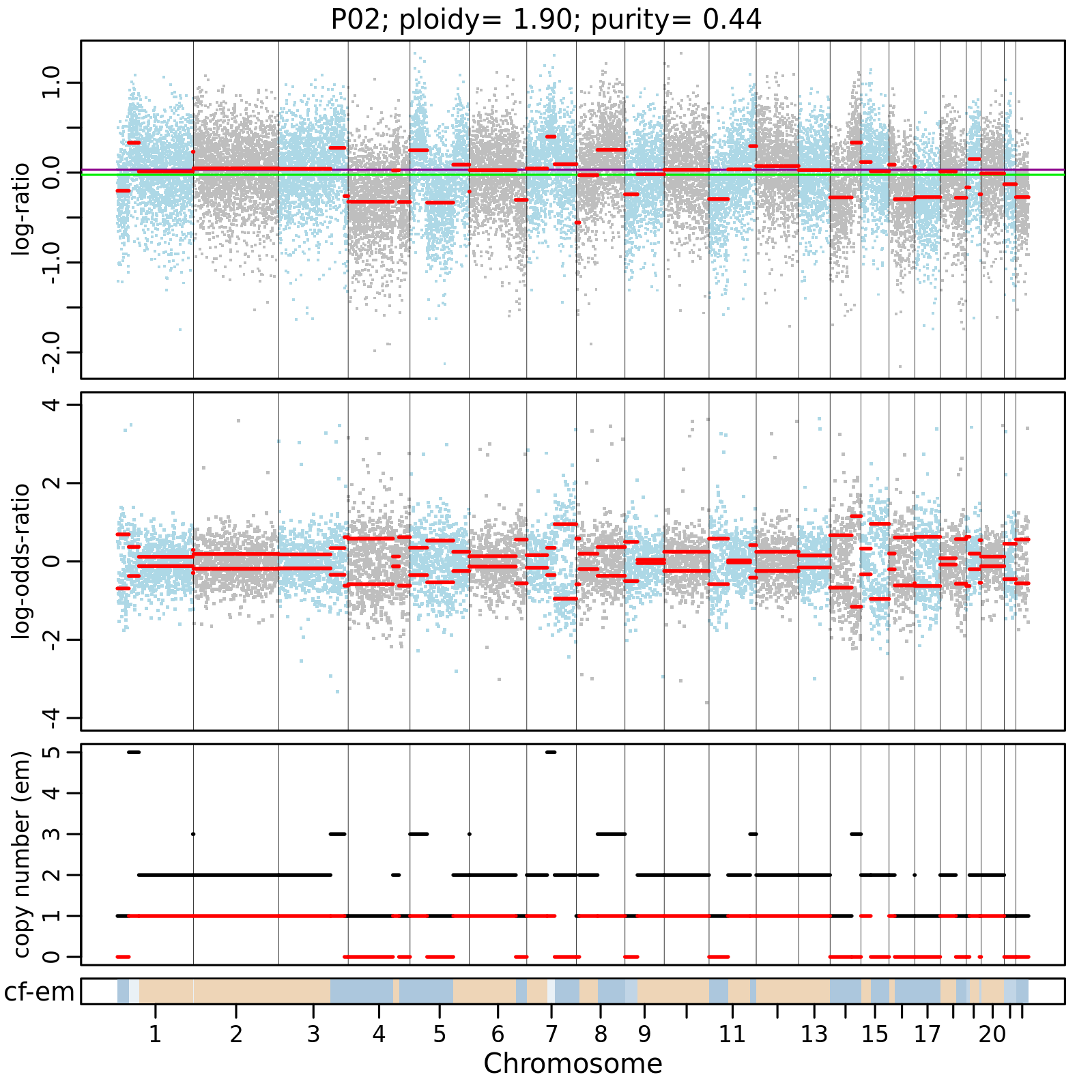

Supplement: Supplementary file 1 [file DataSheet1.ZIP › CNV_plot/P02.cnv.png]

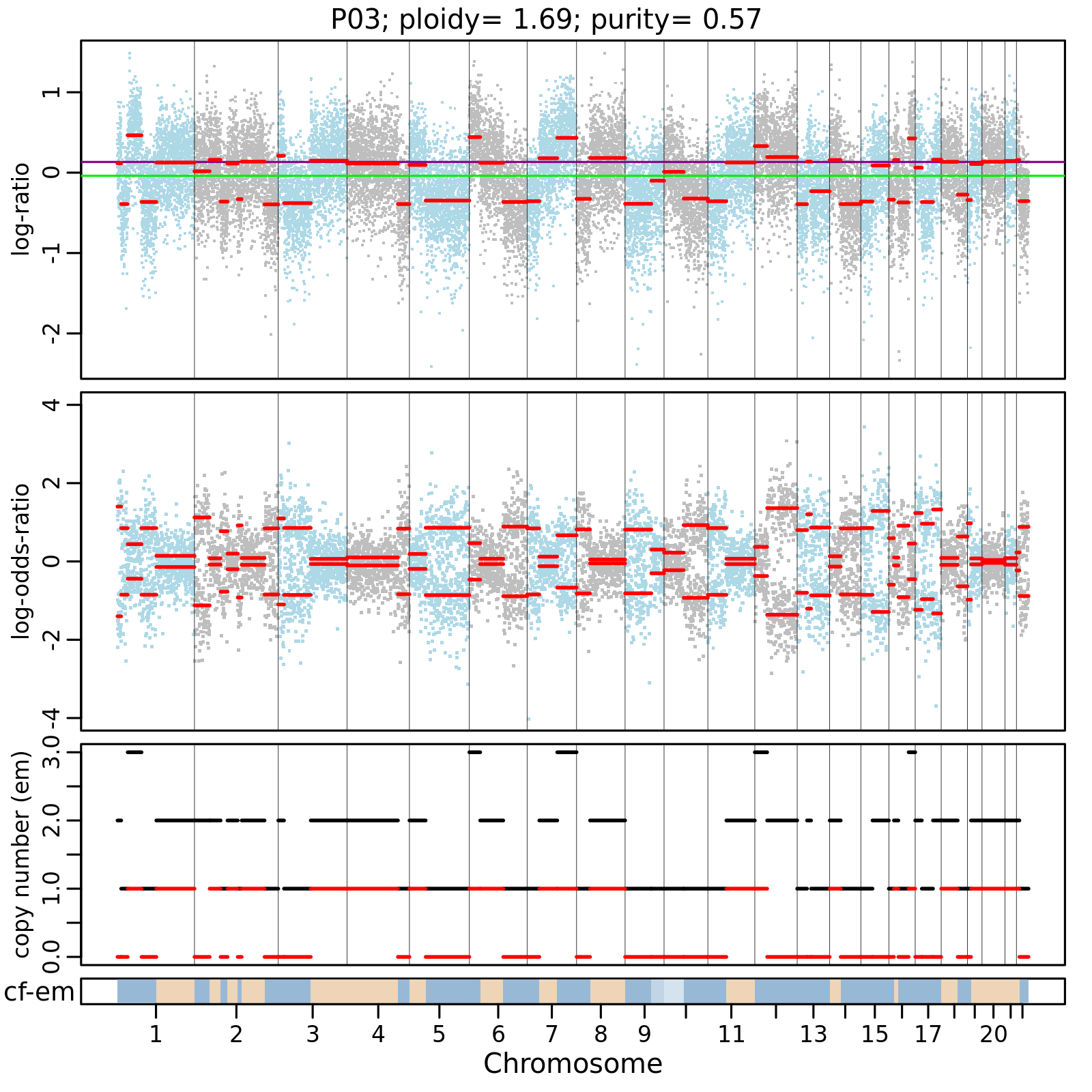

Supplement: Supplementary file 1 [file DataSheet1.ZIP › CNV_plot/P03.cnv.png]

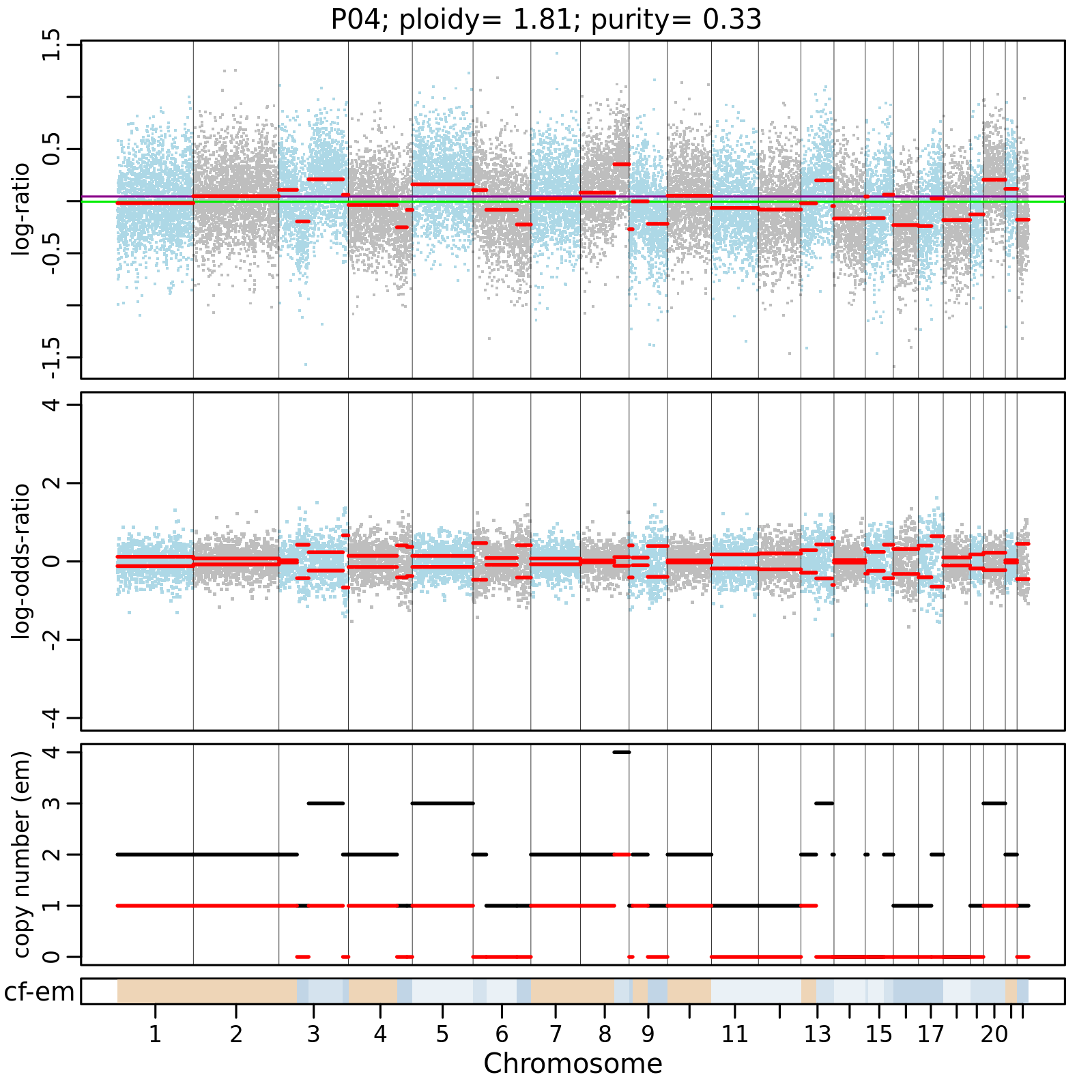

Supplement: Supplementary file 1 [file DataSheet1.ZIP › CNV_plot/P04.cnv.png]

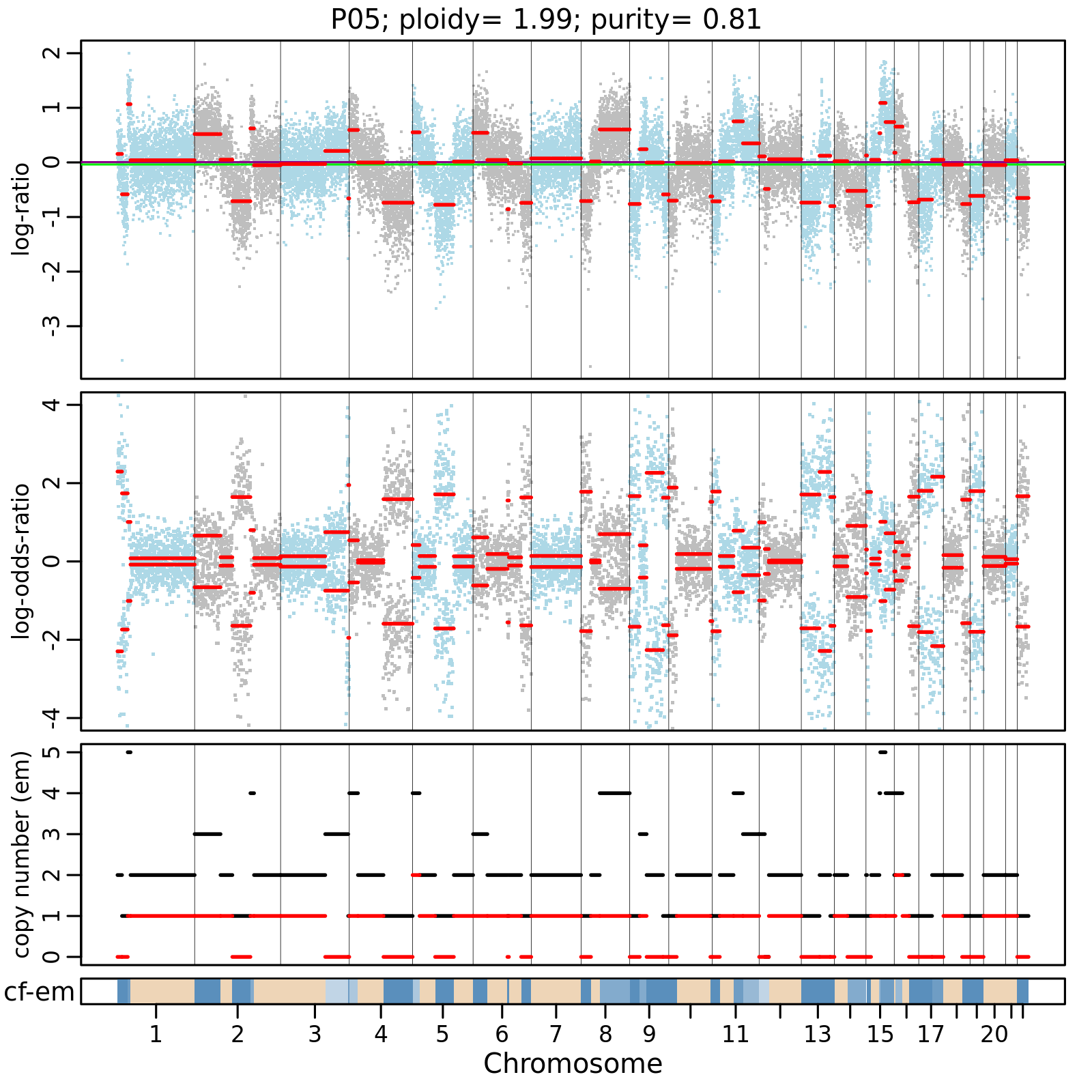

Supplement: Supplementary file 1 [file DataSheet1.ZIP › CNV_plot/P05.cnv.png]

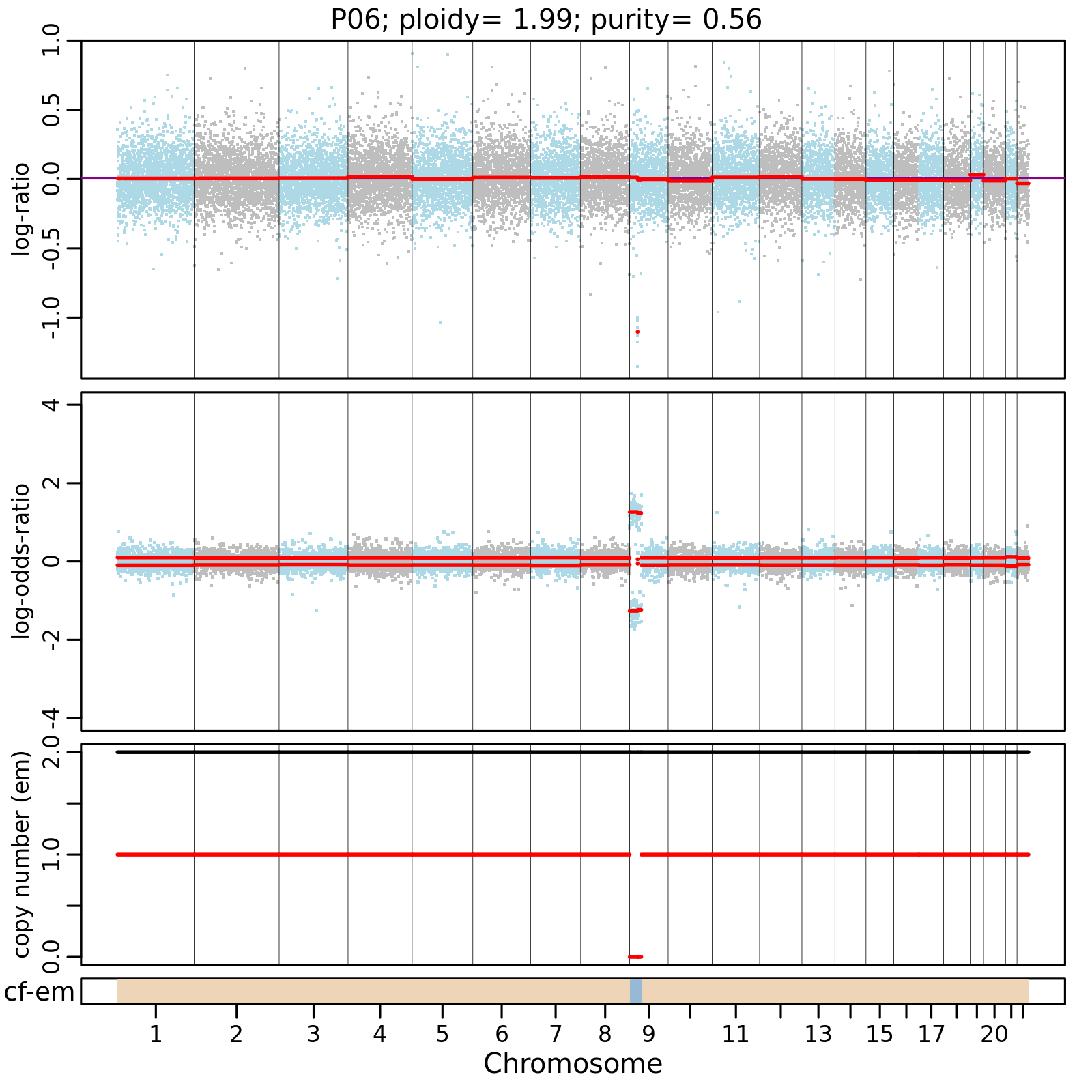

Supplement: Supplementary file 1 [file DataSheet1.ZIP › CNV_plot/P06.cnv.png]

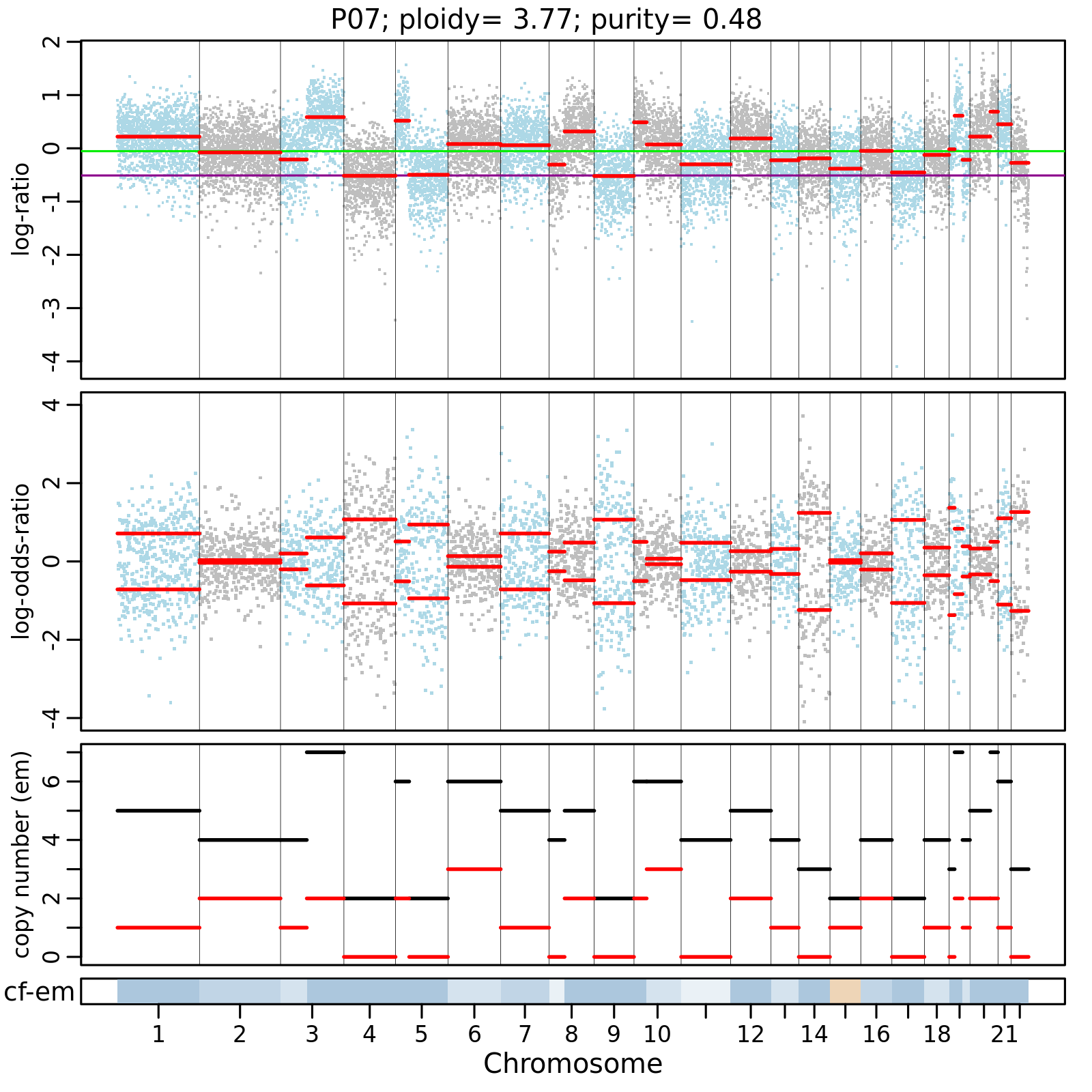

Supplement: Supplementary file 1 [file DataSheet1.ZIP › CNV_plot/P07.cnv.png]

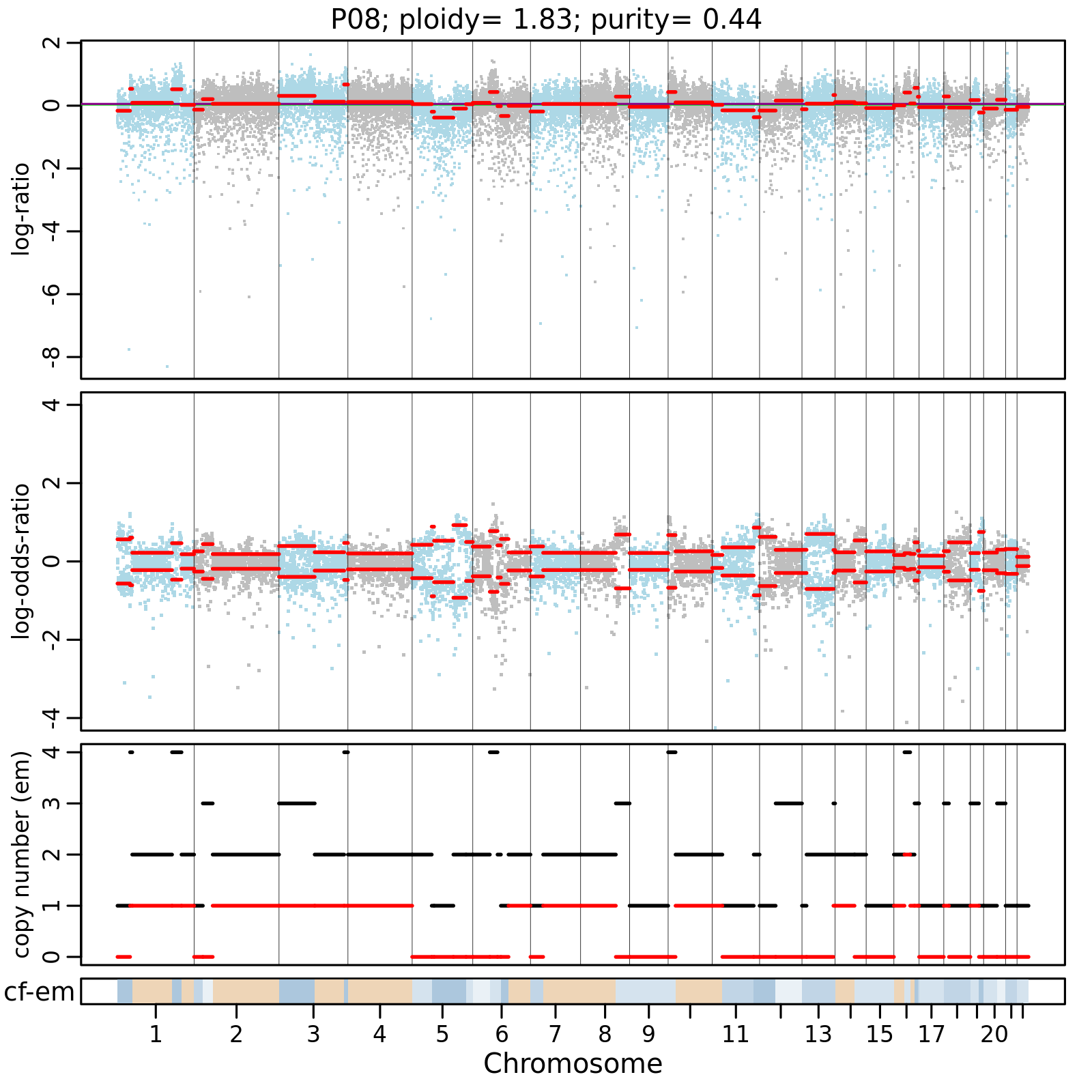

Supplement: Supplementary file 1 [file DataSheet1.ZIP › CNV_plot/P08.cnv.png]

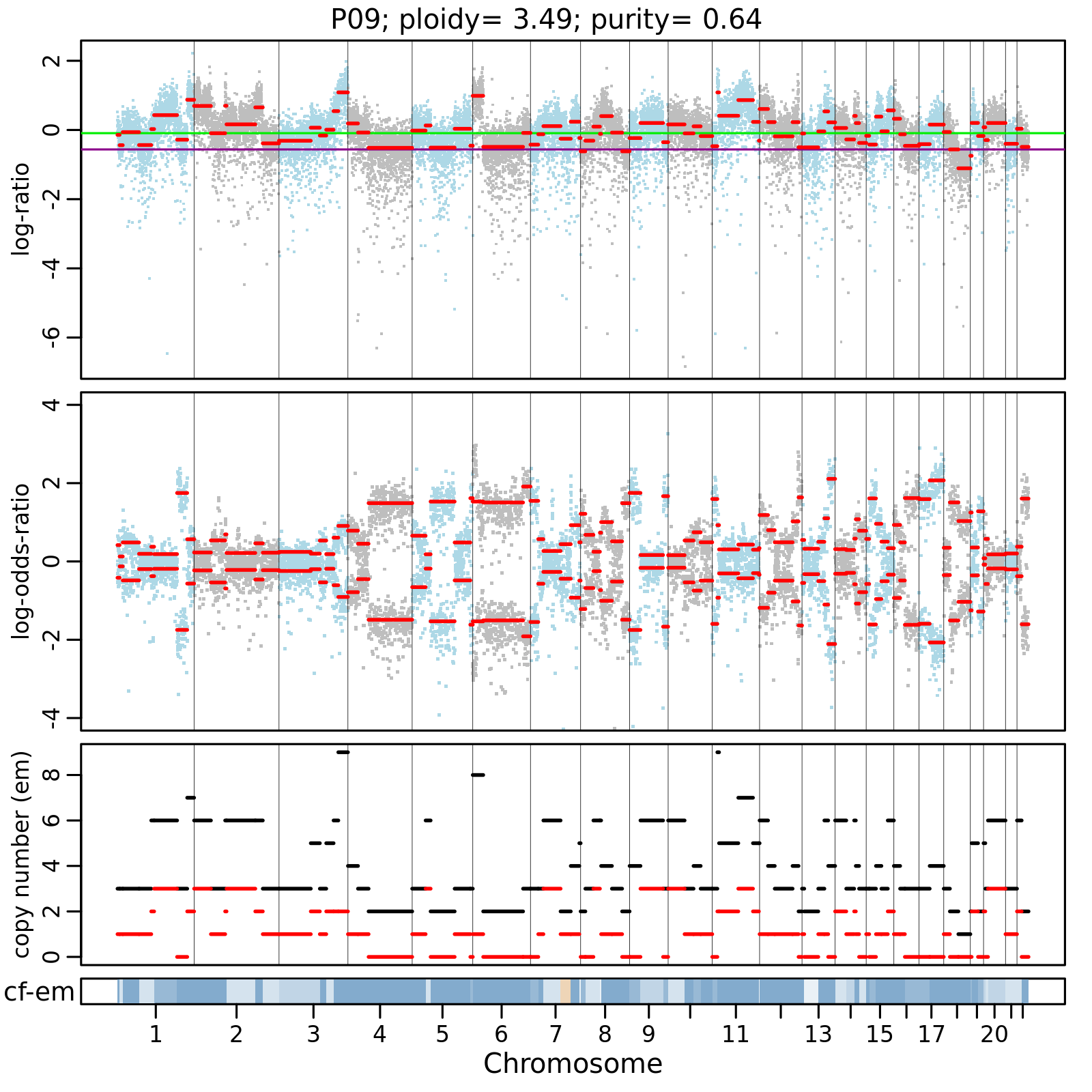

Supplement: Supplementary file 1 [file DataSheet1.ZIP › CNV_plot/P09.cnv.png]

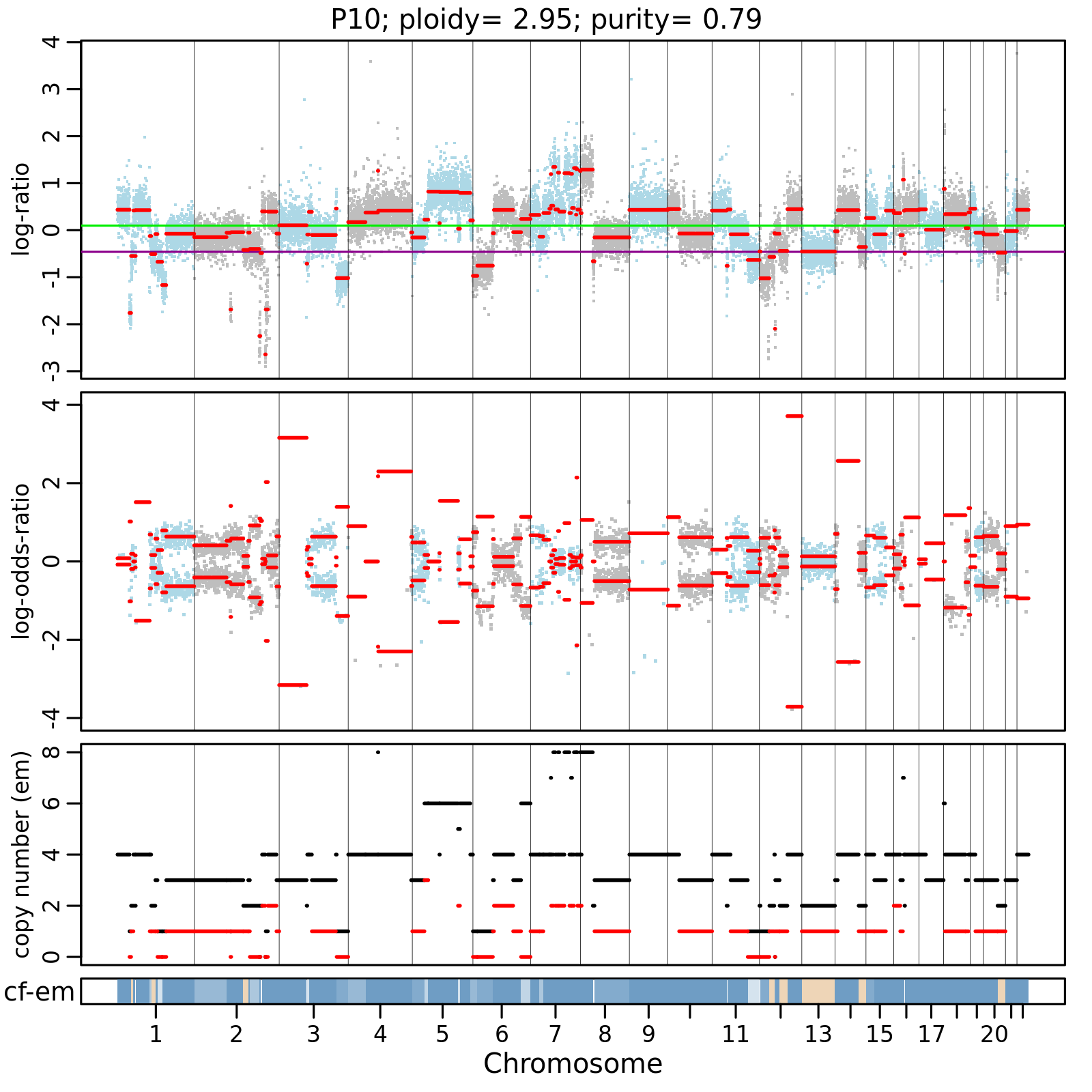

Supplement: Supplementary file 1 [file DataSheet1.ZIP › CNV_plot/P10.cnv.png]

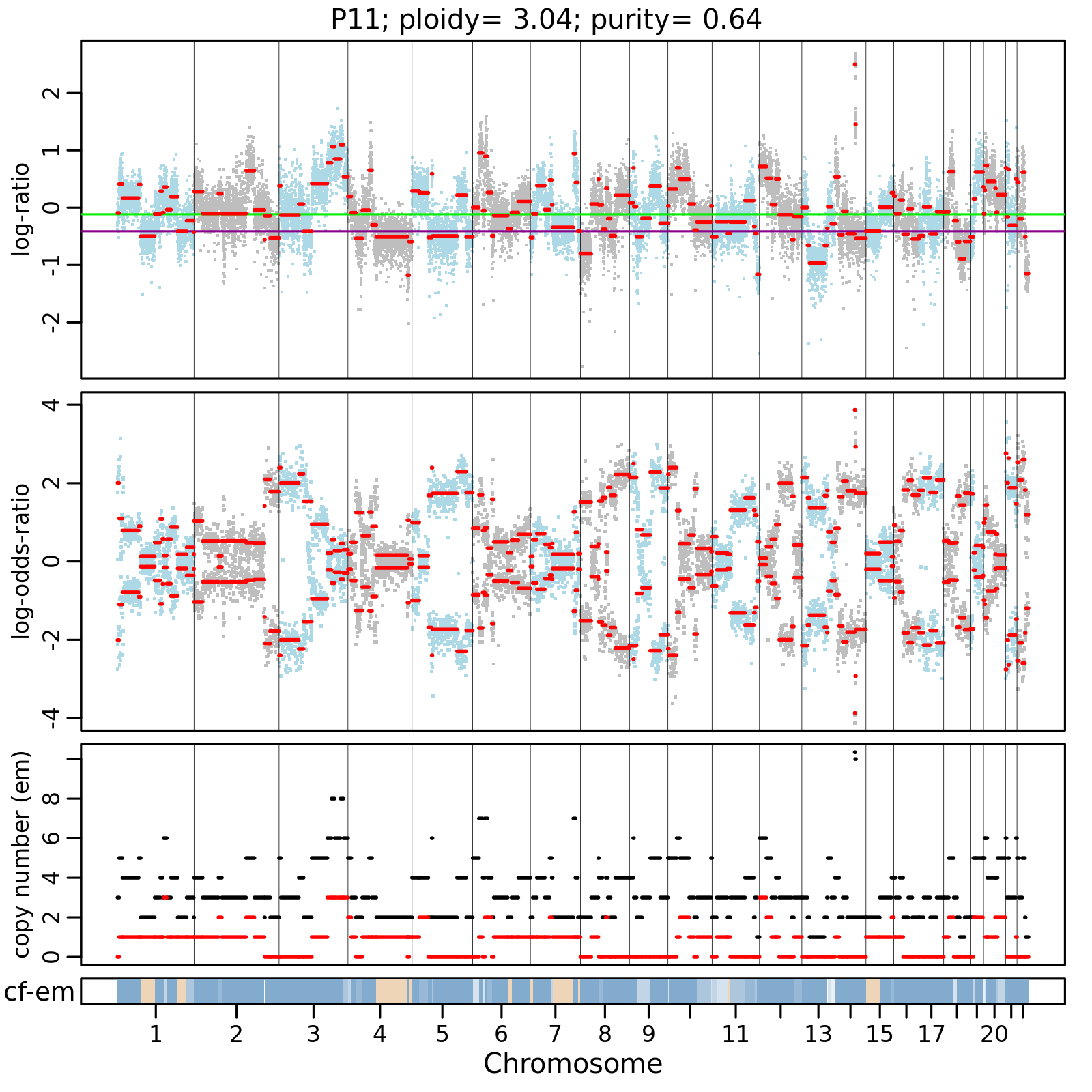

Supplement: Supplementary file 1 [file DataSheet1.ZIP › CNV_plot/P11.cnv.png]

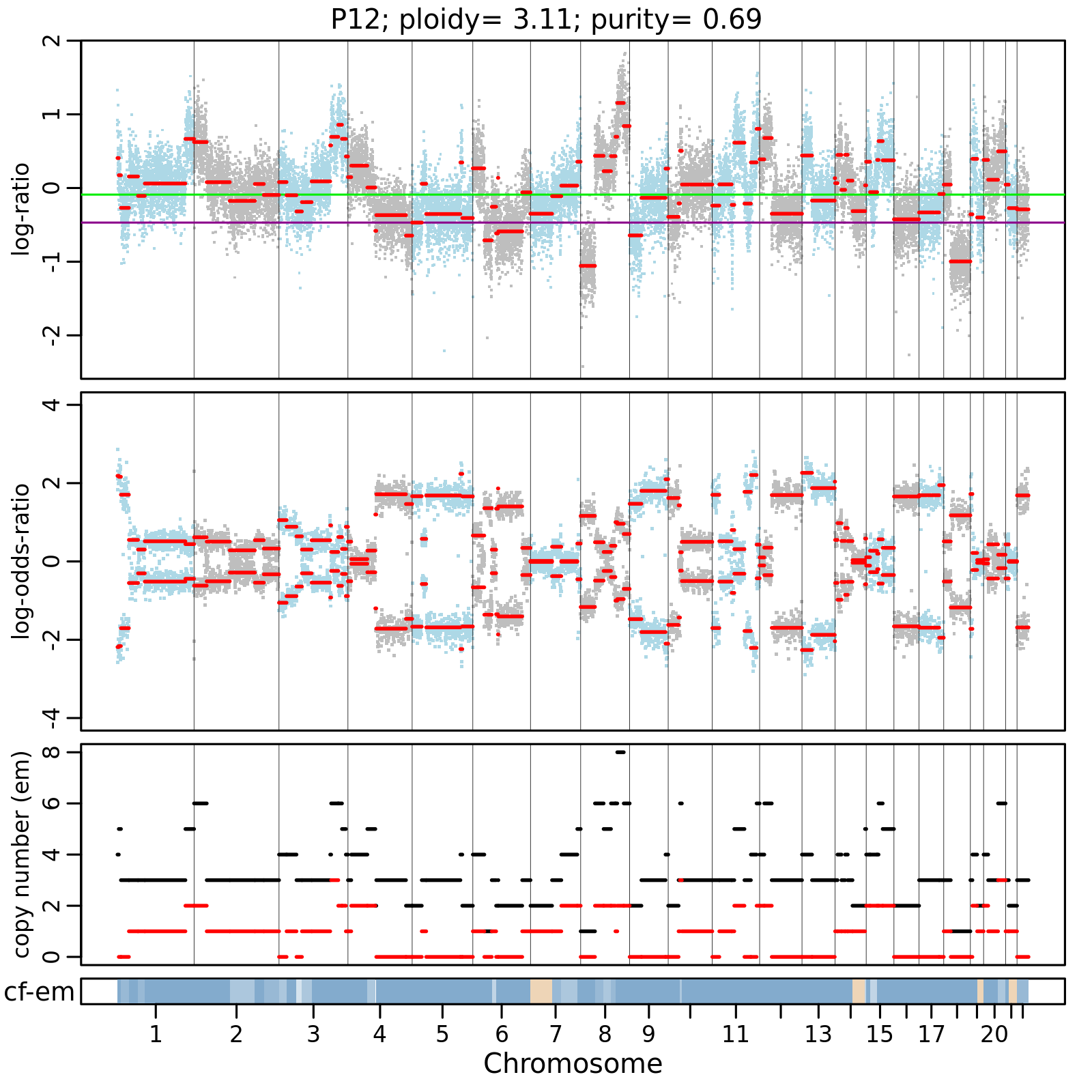

Supplement: Supplementary file 1 [file DataSheet1.ZIP › CNV_plot/P12.cnv.png]

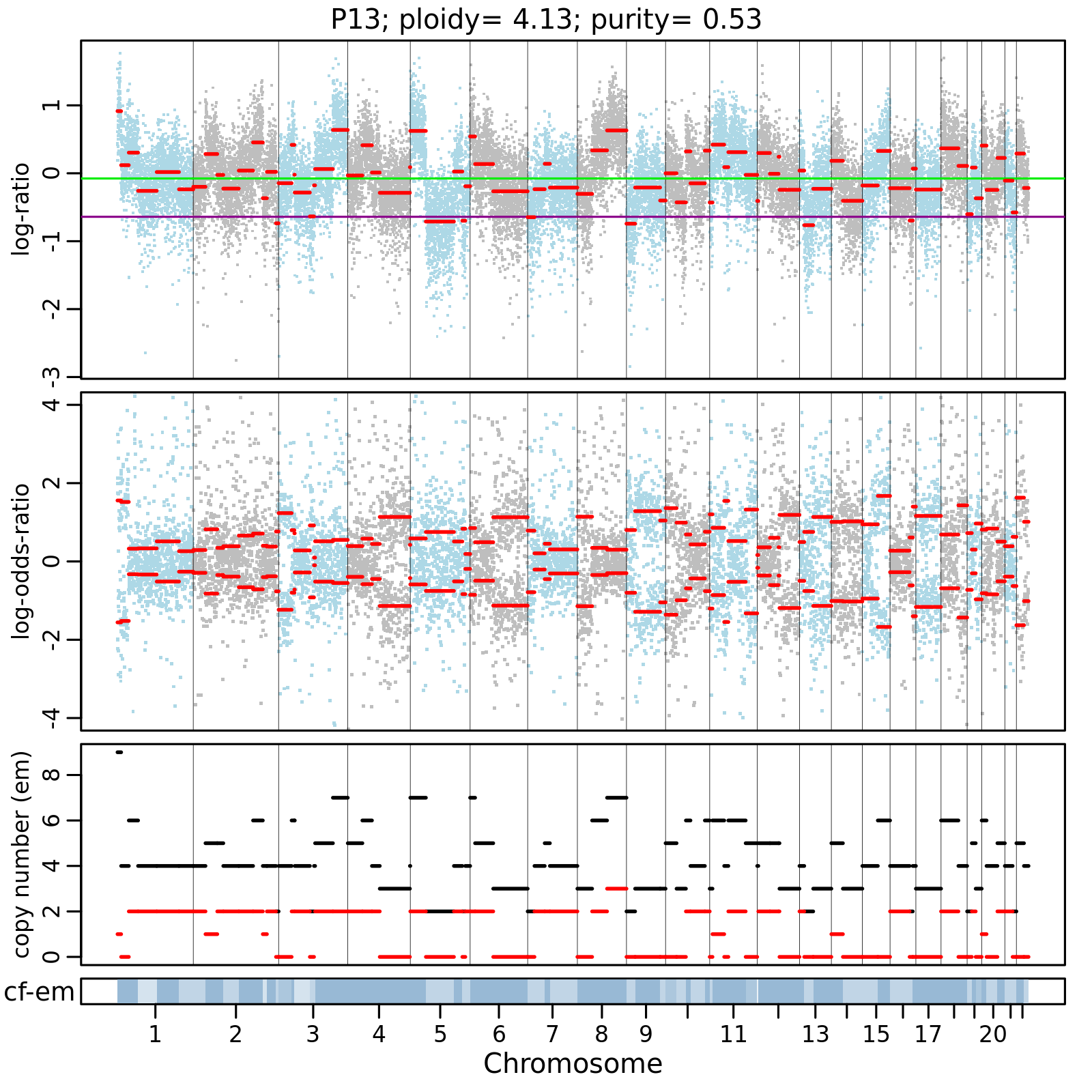

Supplement: Supplementary file 1 [file DataSheet1.ZIP › CNV_plot/P13.cnv.png]

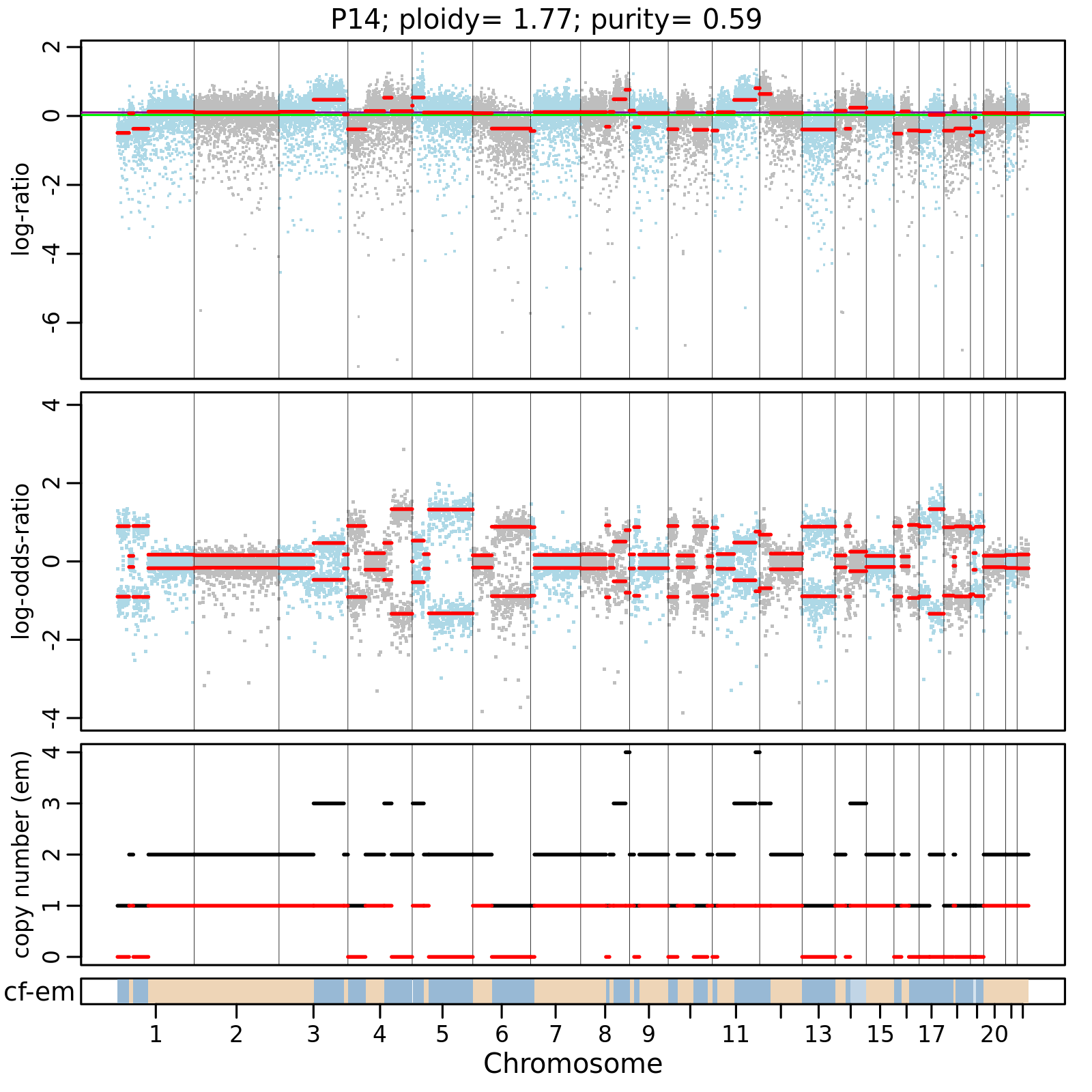

Supplement: Supplementary file 1 [file DataSheet1.ZIP › CNV_plot/P14.cnv.png]

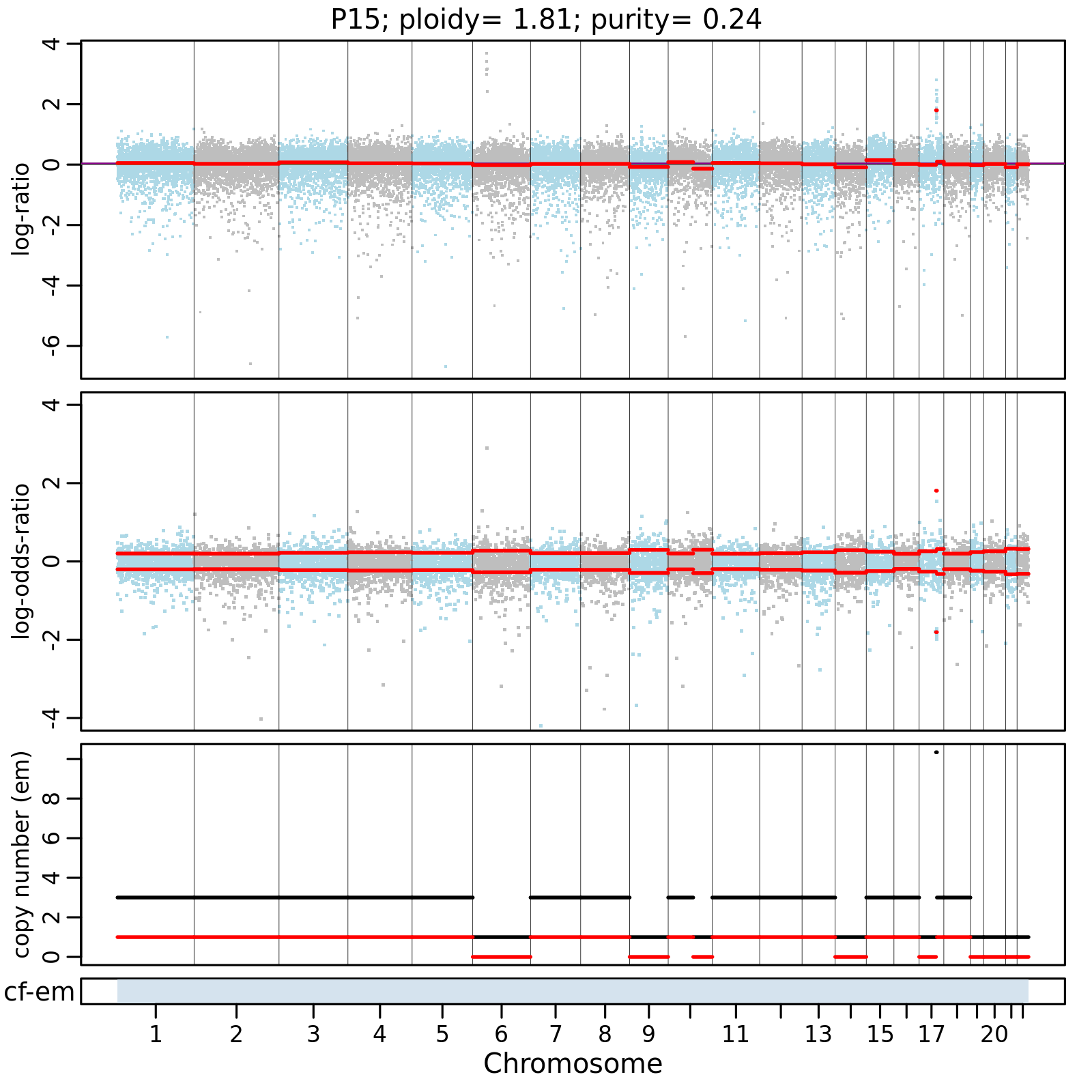

Supplement: Supplementary file 1 [file DataSheet1.ZIP › CNV_plot/P15.cnv.png]

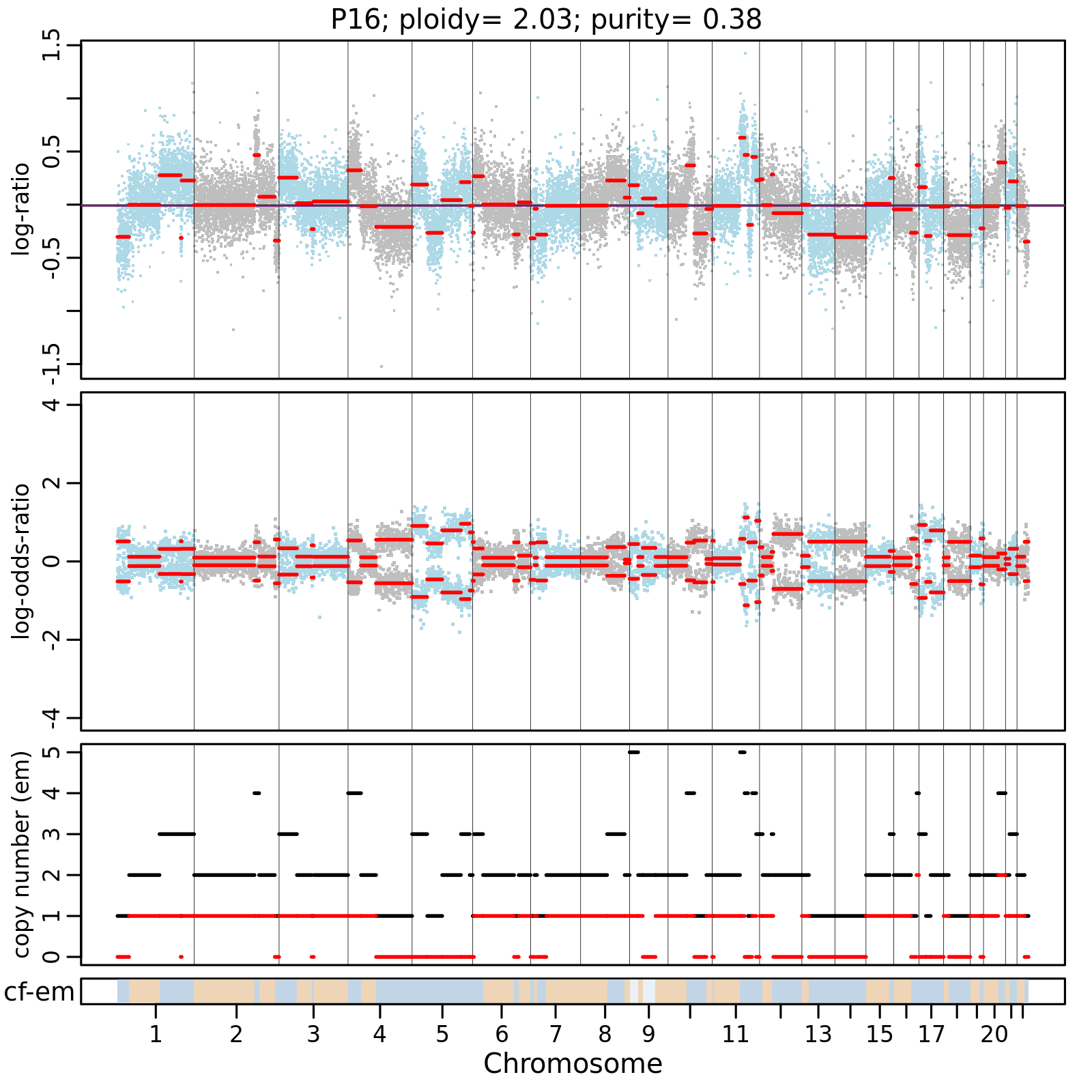

Supplement: Supplementary file 1 [file DataSheet1.ZIP › CNV_plot/P16.cnv.png]

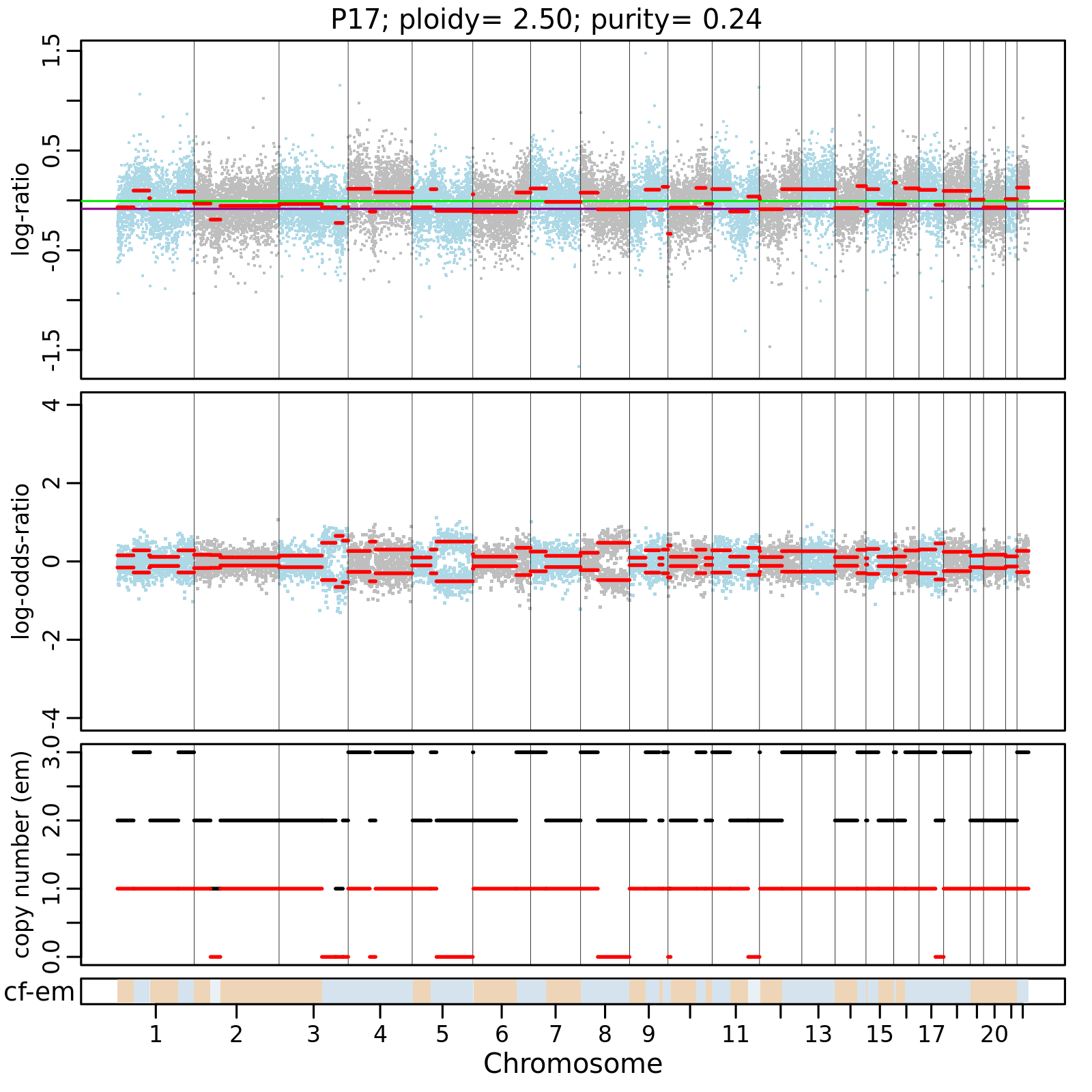

Supplement: Supplementary file 1 [file DataSheet1.ZIP › CNV_plot/P17.cnv.png]

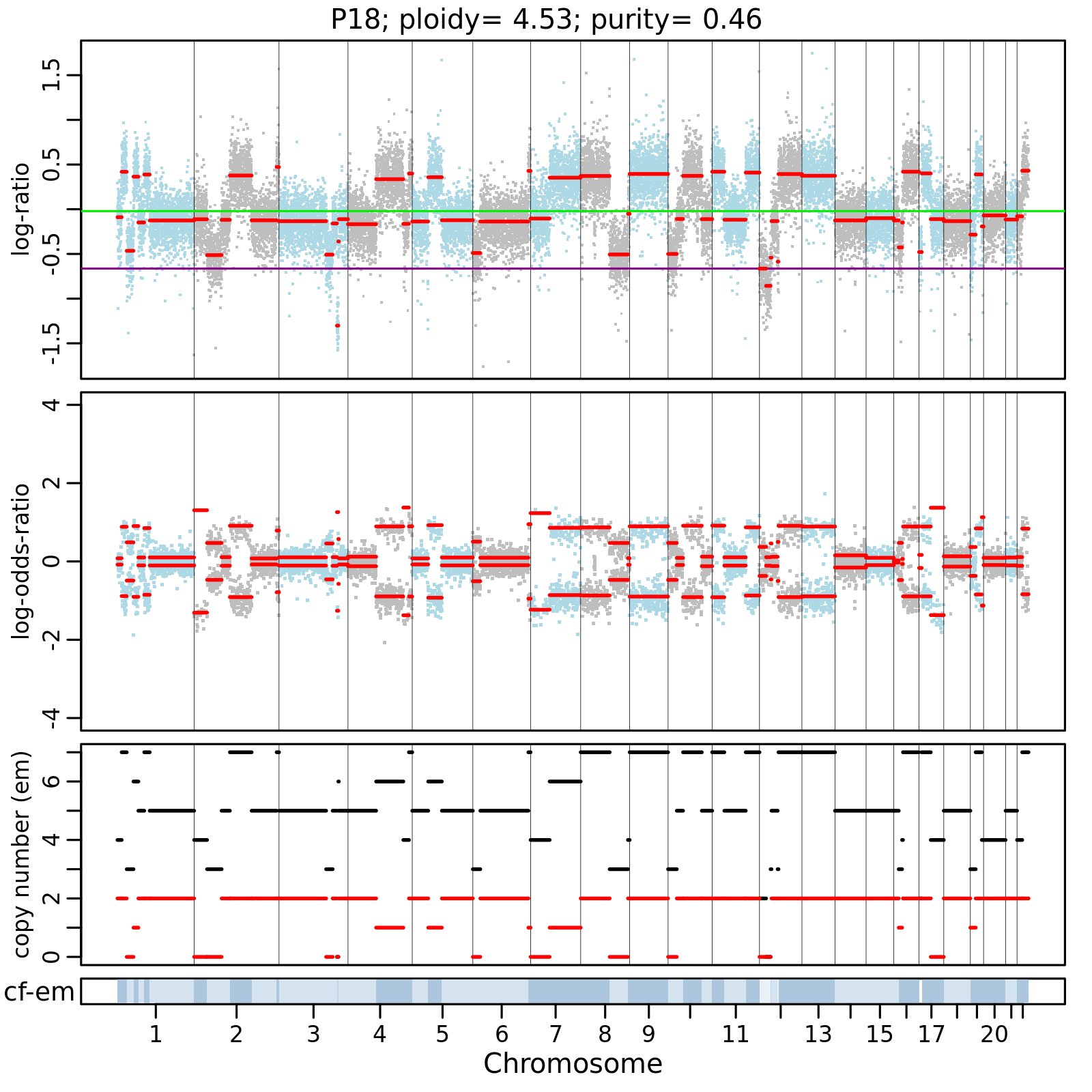

Supplement: Supplementary file 1 [file DataSheet1.ZIP › CNV_plot/P18.cnv.png]

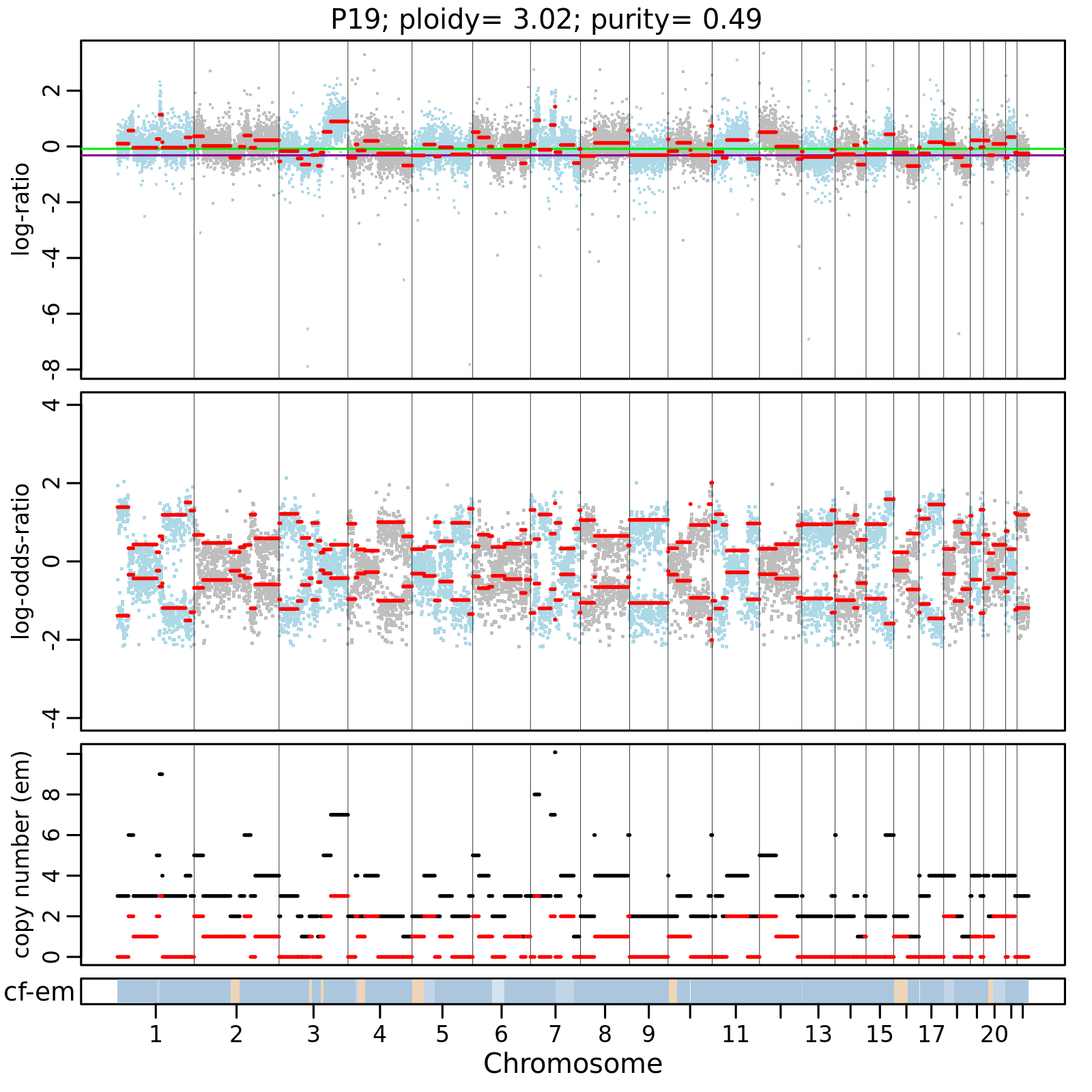

Supplement: Supplementary file 1 [file DataSheet1.ZIP › CNV_plot/P19.cnv.png]

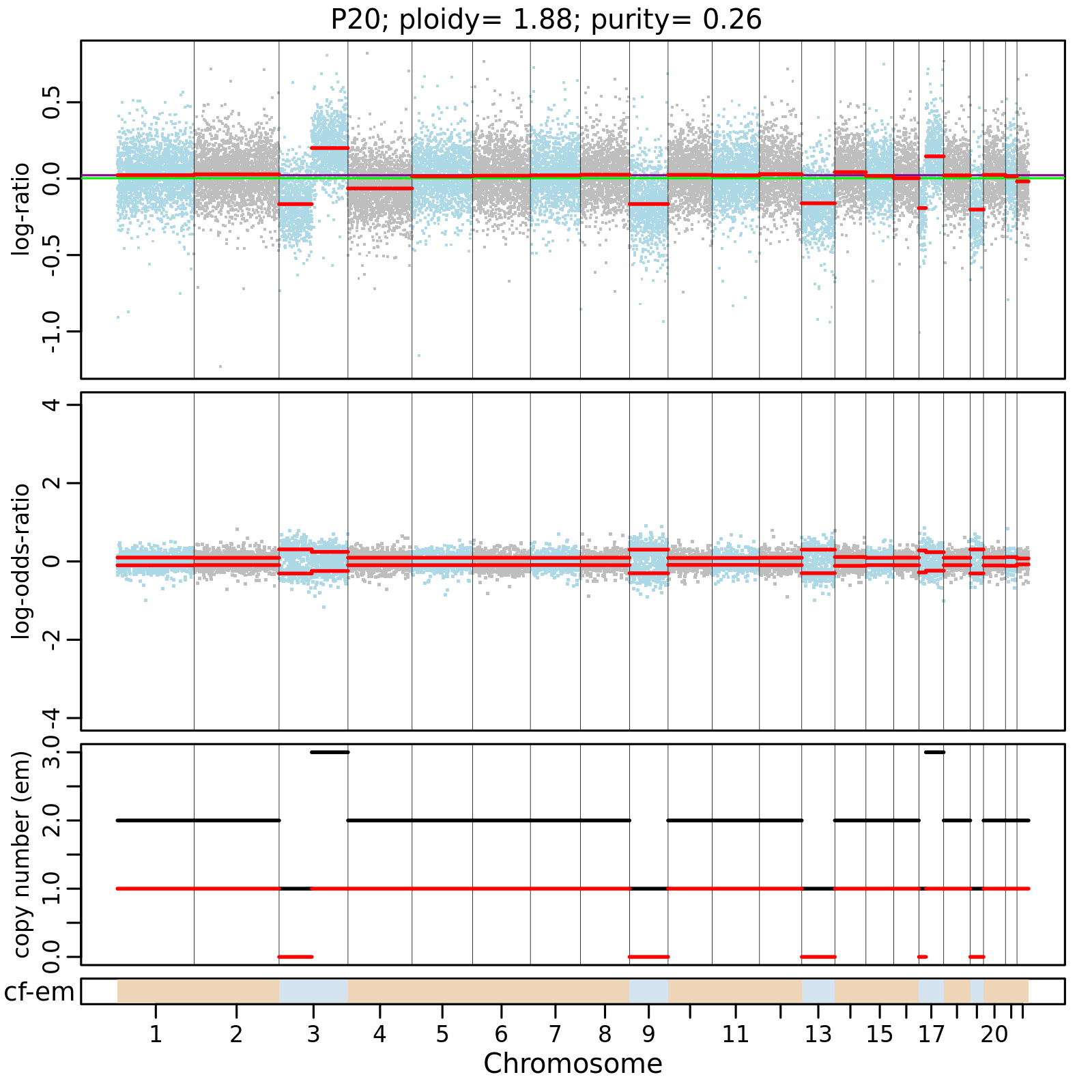

Supplement: Supplementary file 1 [file DataSheet1.ZIP › CNV_plot/P20.cnv.png]

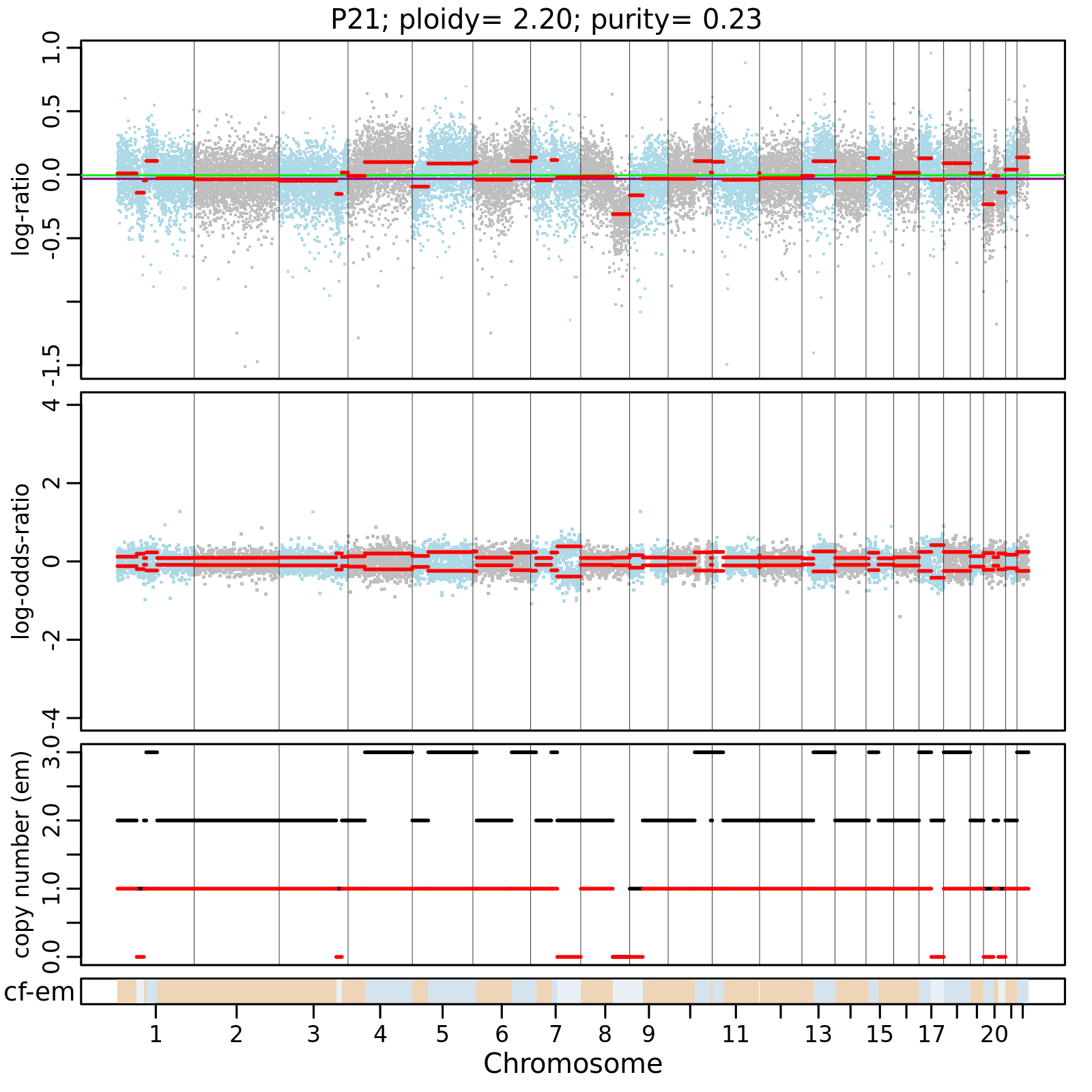

Supplement: Supplementary file 1 [file DataSheet1.ZIP › CNV_plot/P21.cnv.png]

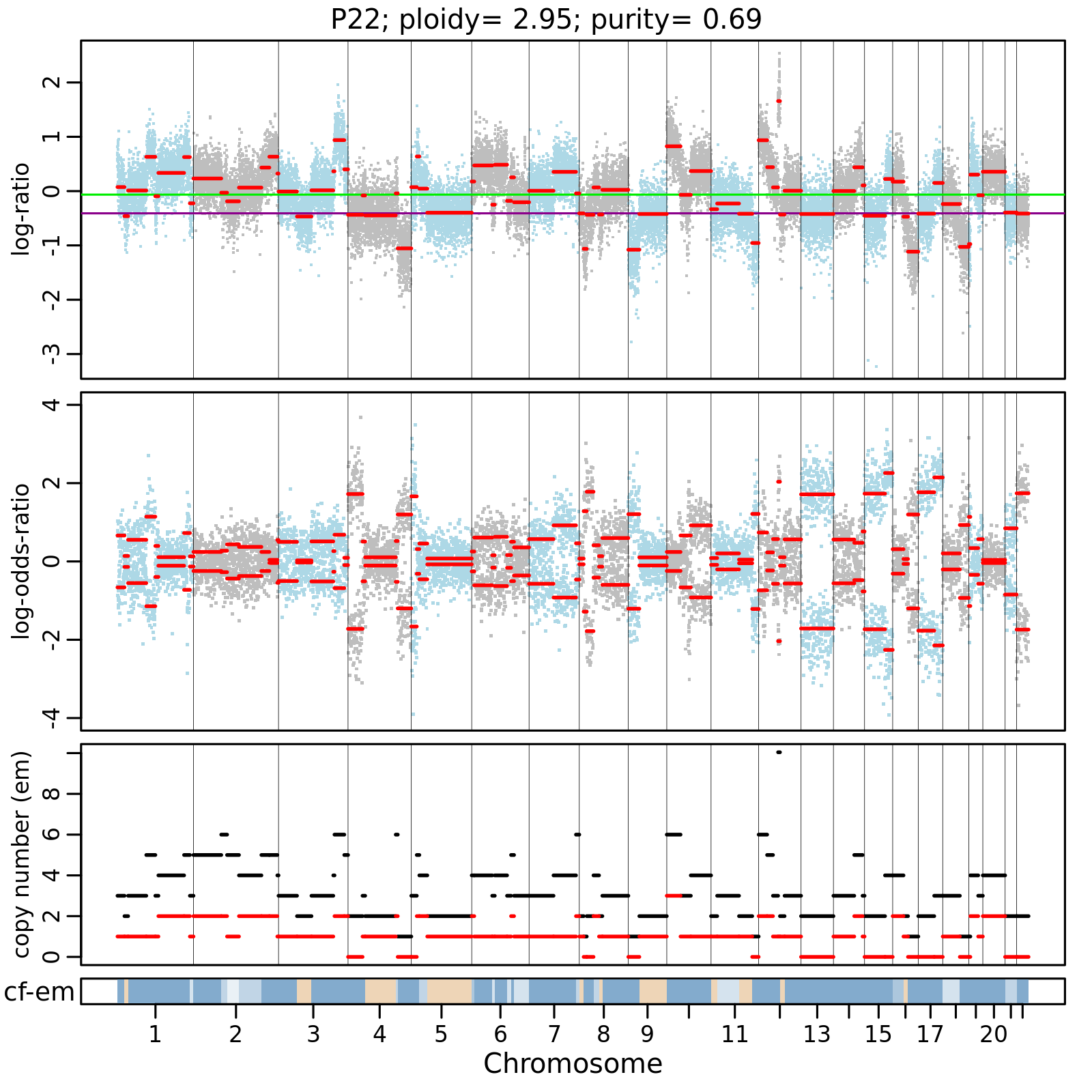

Supplement: Supplementary file 1 [file DataSheet1.ZIP › CNV_plot/P22.cnv.png]

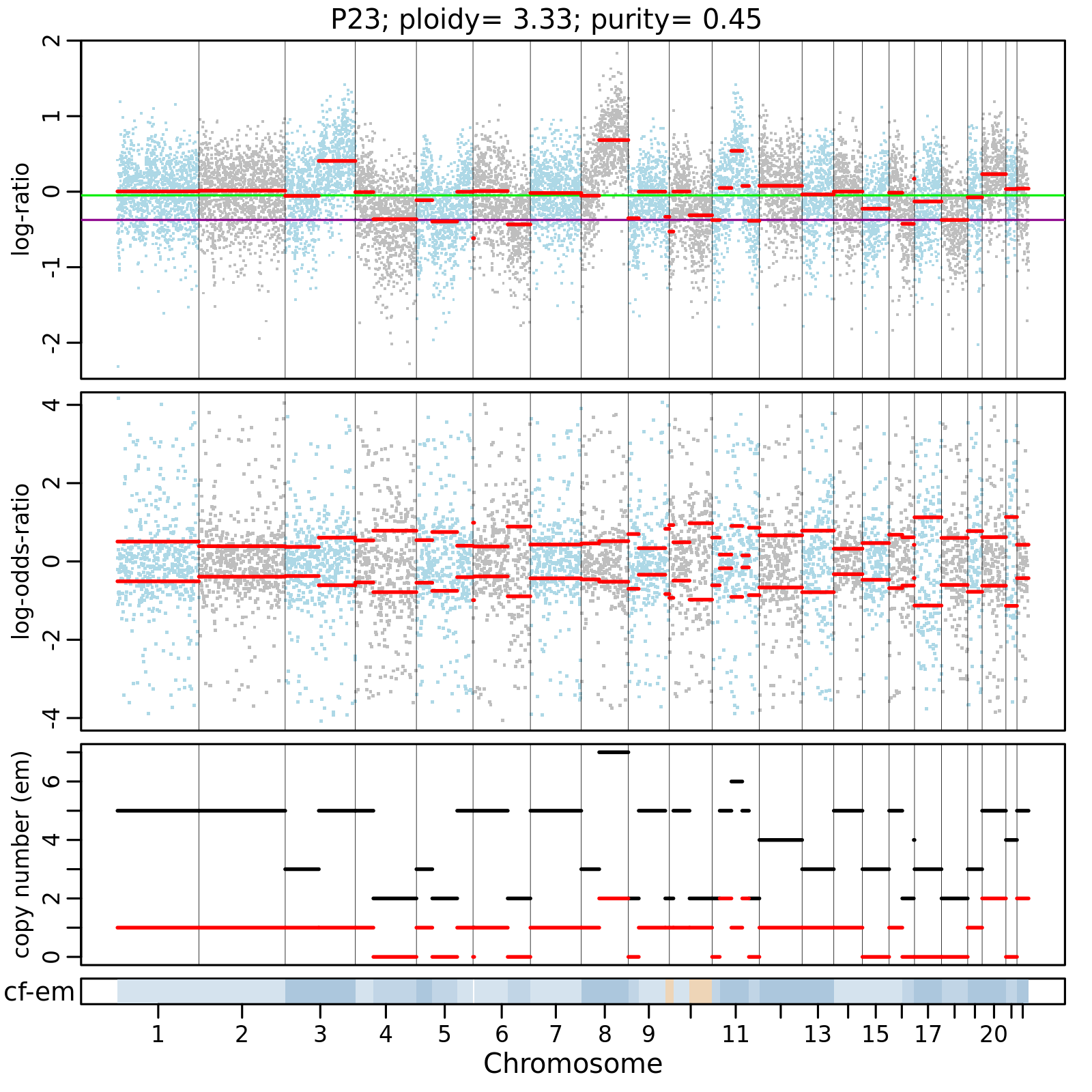

Supplement: Supplementary file 1 [file DataSheet1.ZIP › CNV_plot/P23.cnv.png]

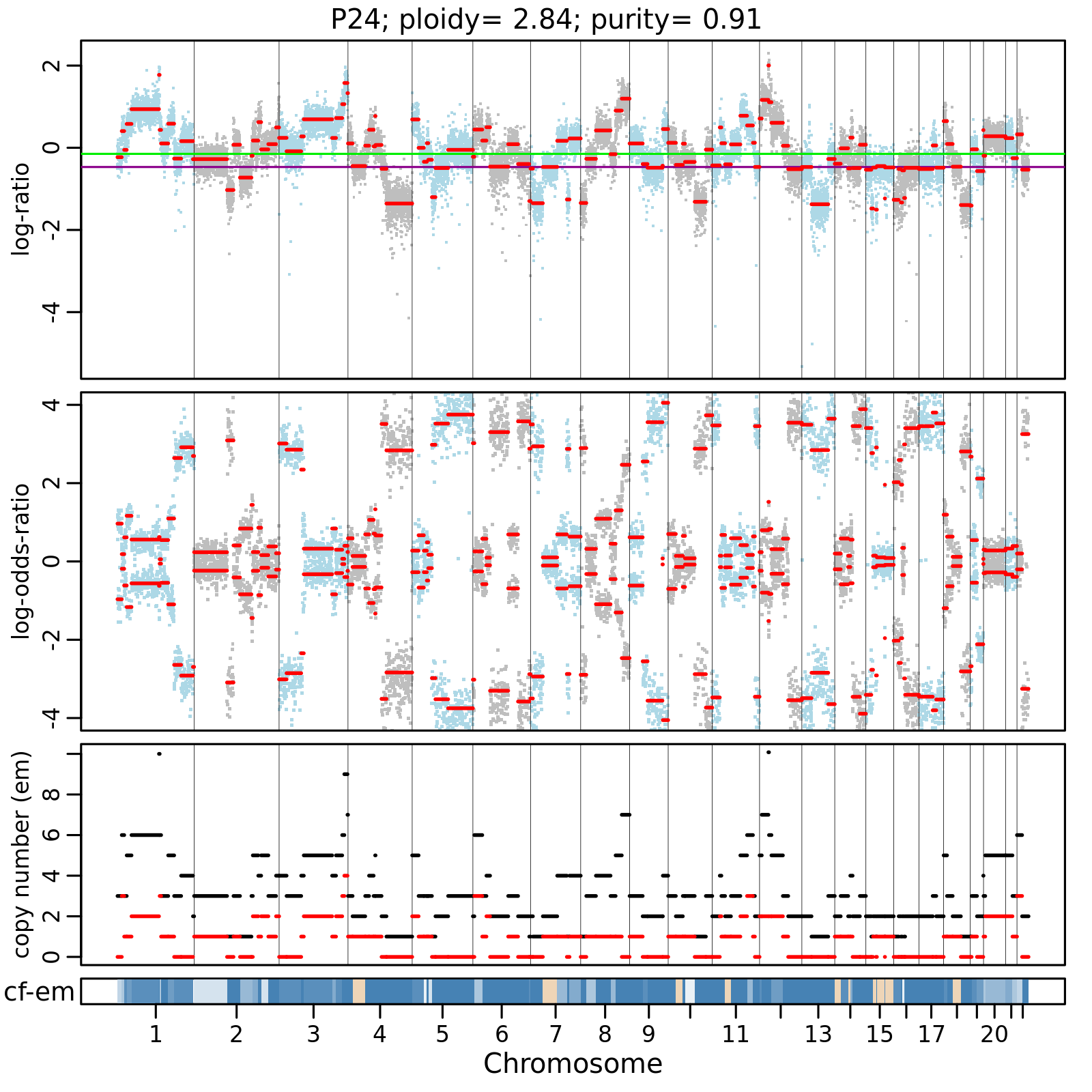

Supplement: Supplementary file 1 [file DataSheet1.ZIP › CNV_plot/P24.cnv.png]

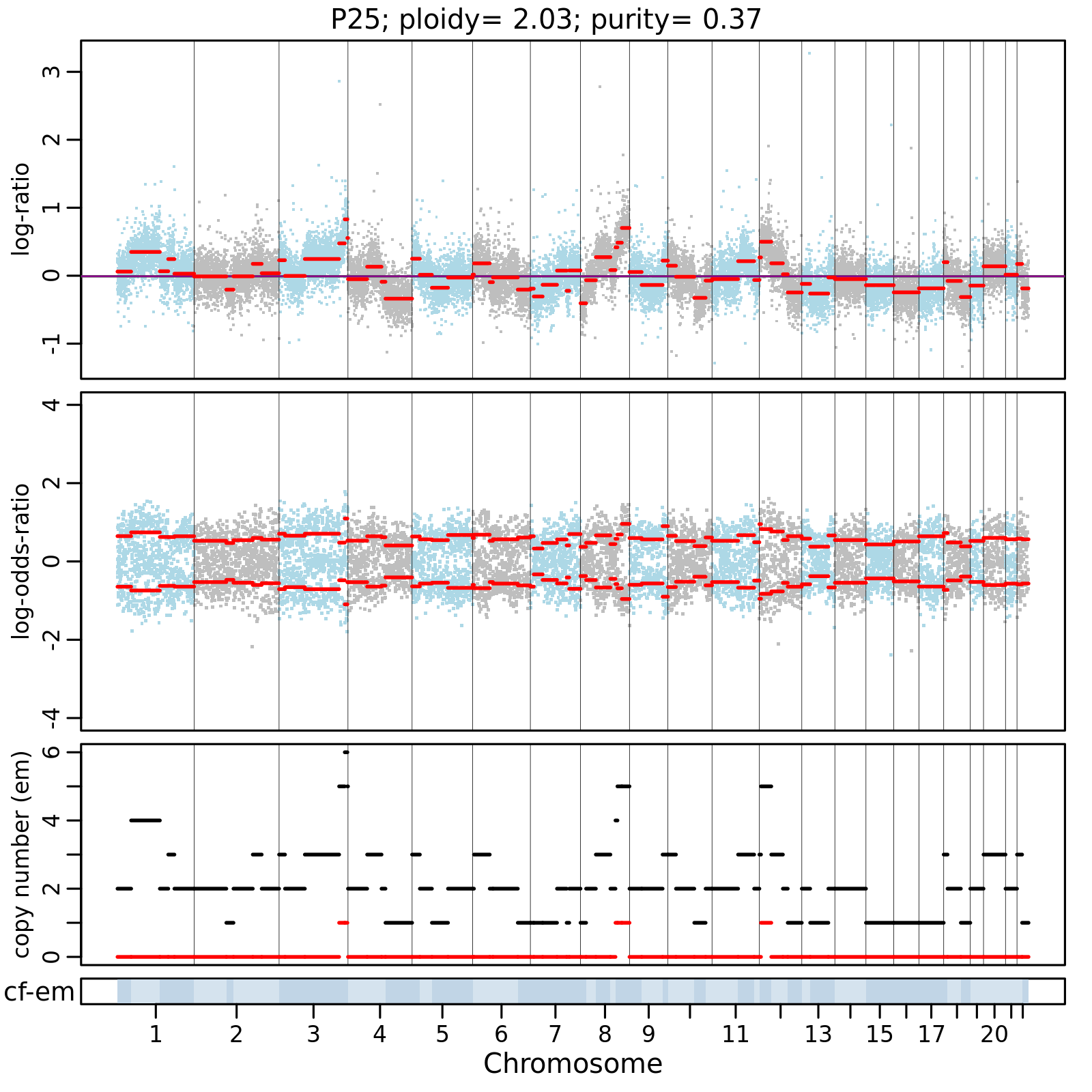

Supplement: Supplementary file 1 [file DataSheet1.ZIP › CNV_plot/P25.cnv.png]

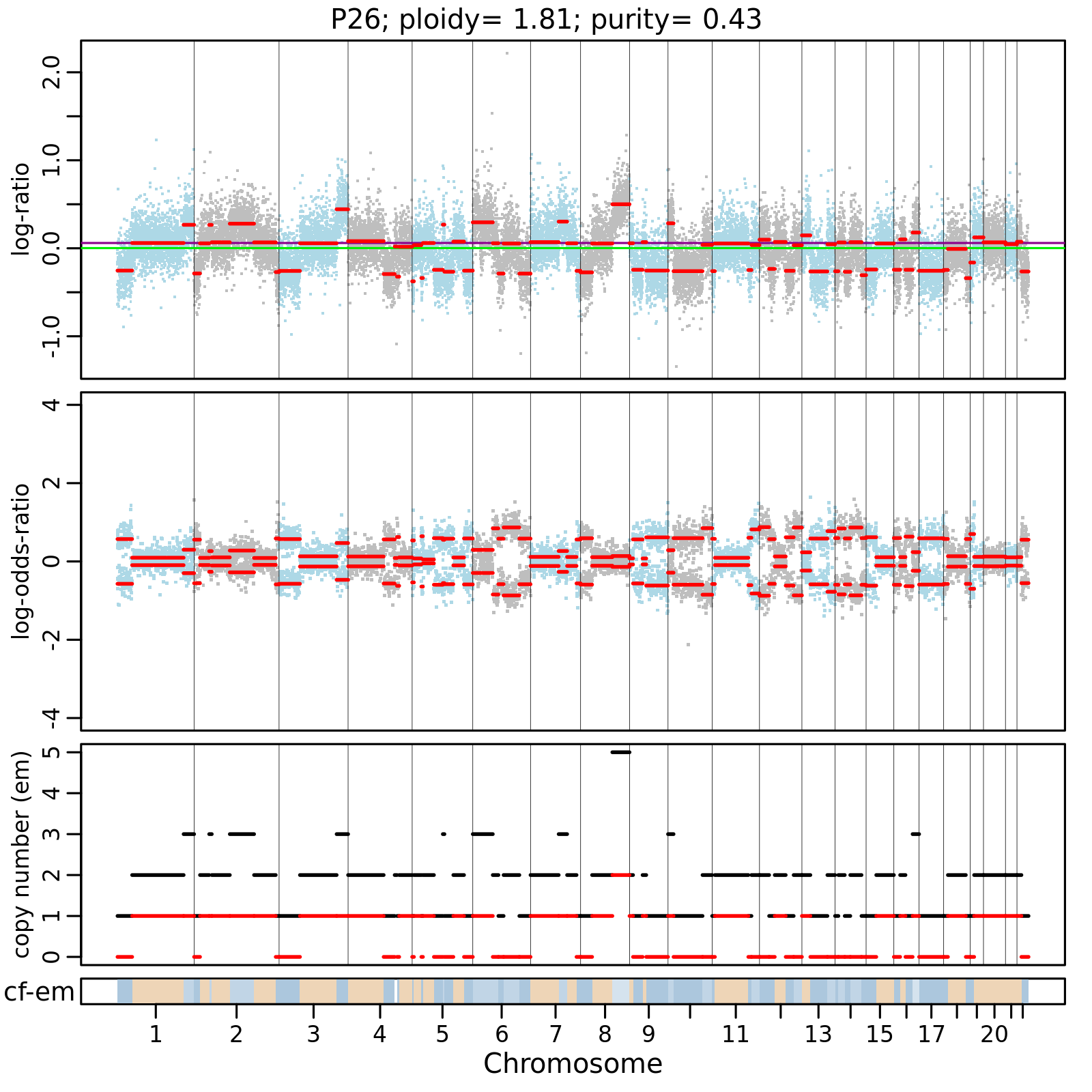

Supplement: Supplementary file 1 [file DataSheet1.ZIP › CNV_plot/P26.cnv.png]

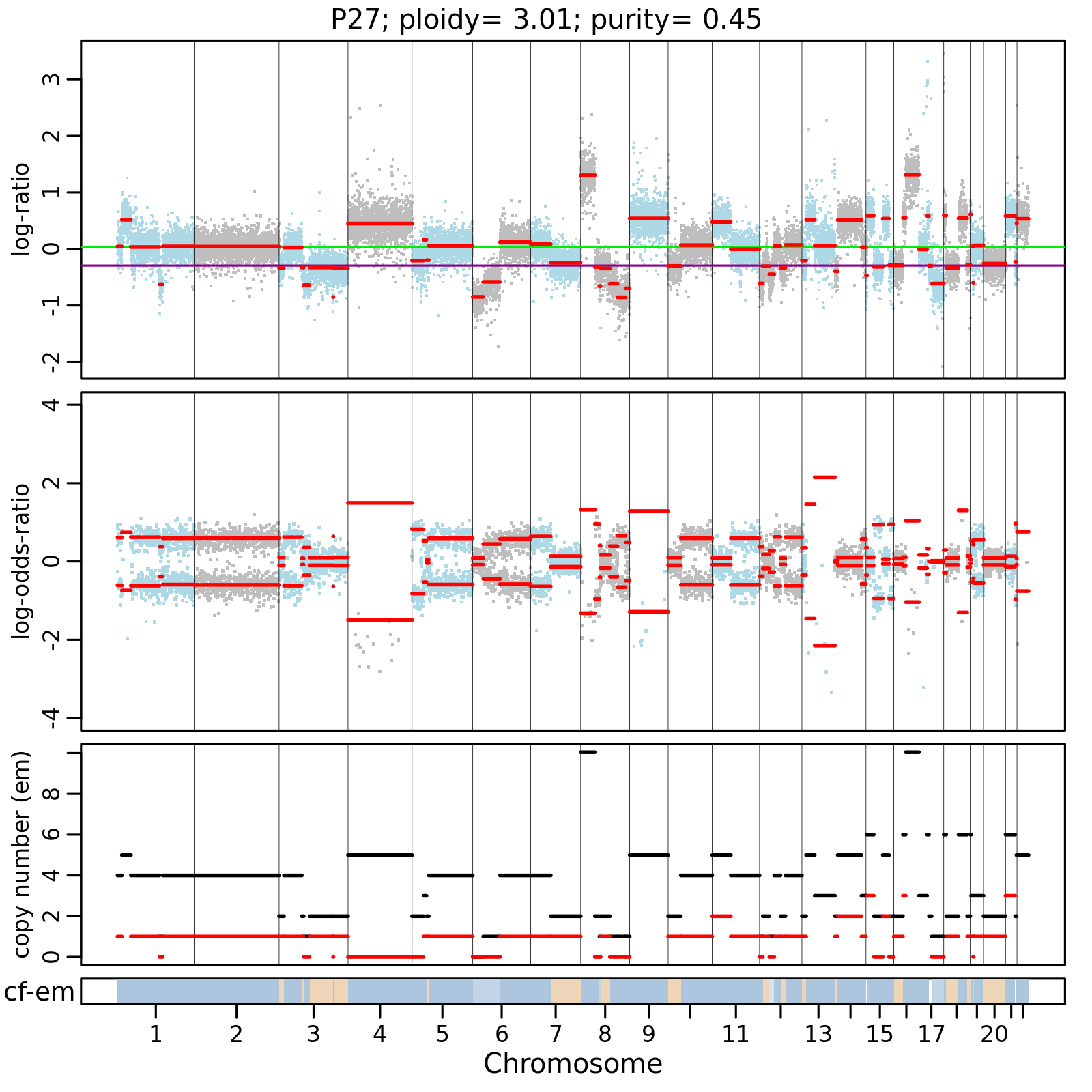

Supplement: Supplementary file 1 [file DataSheet1.ZIP › CNV_plot/P27.cnv.png]

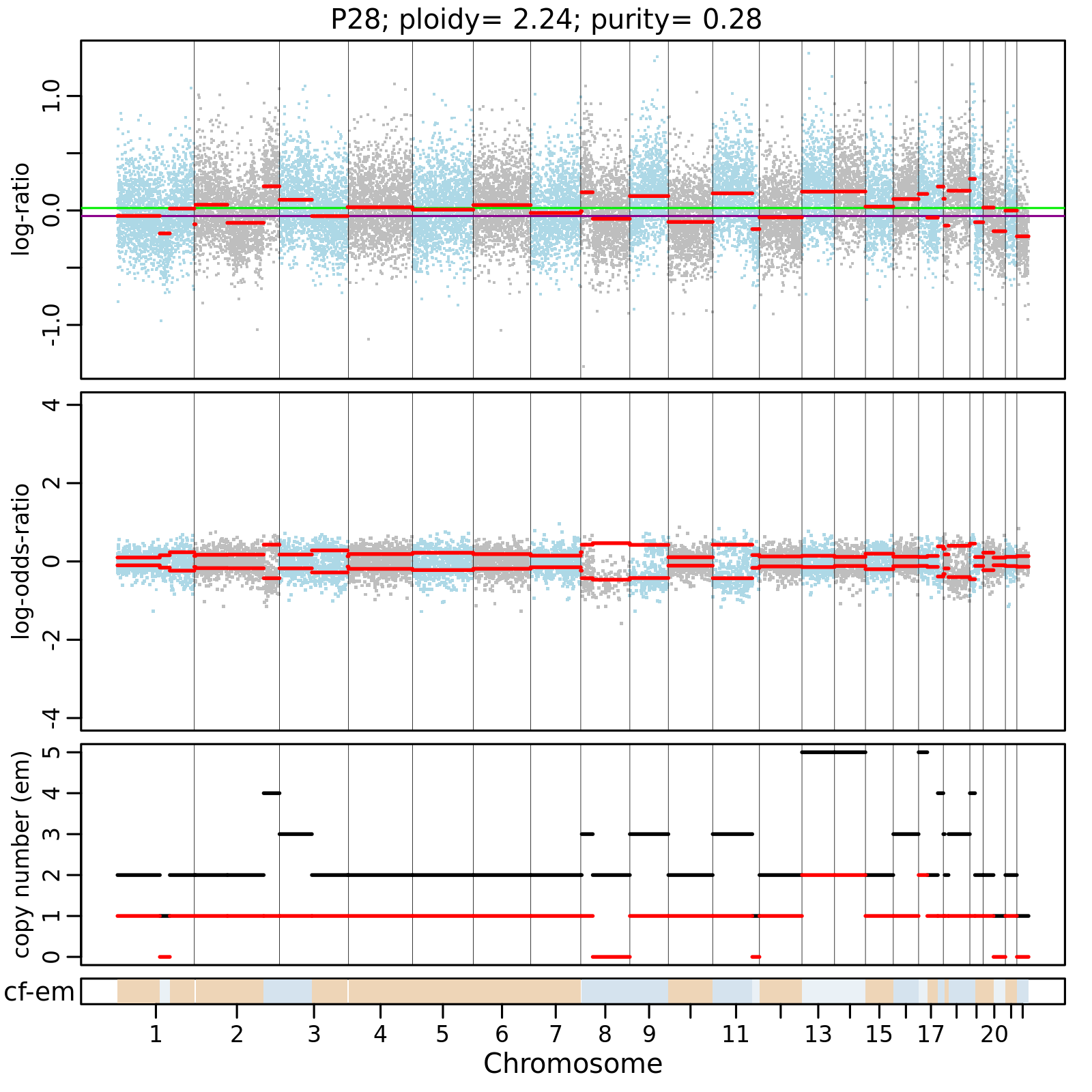

Supplement: Supplementary file 1 [file DataSheet1.ZIP › CNV_plot/P28.cnv.png]

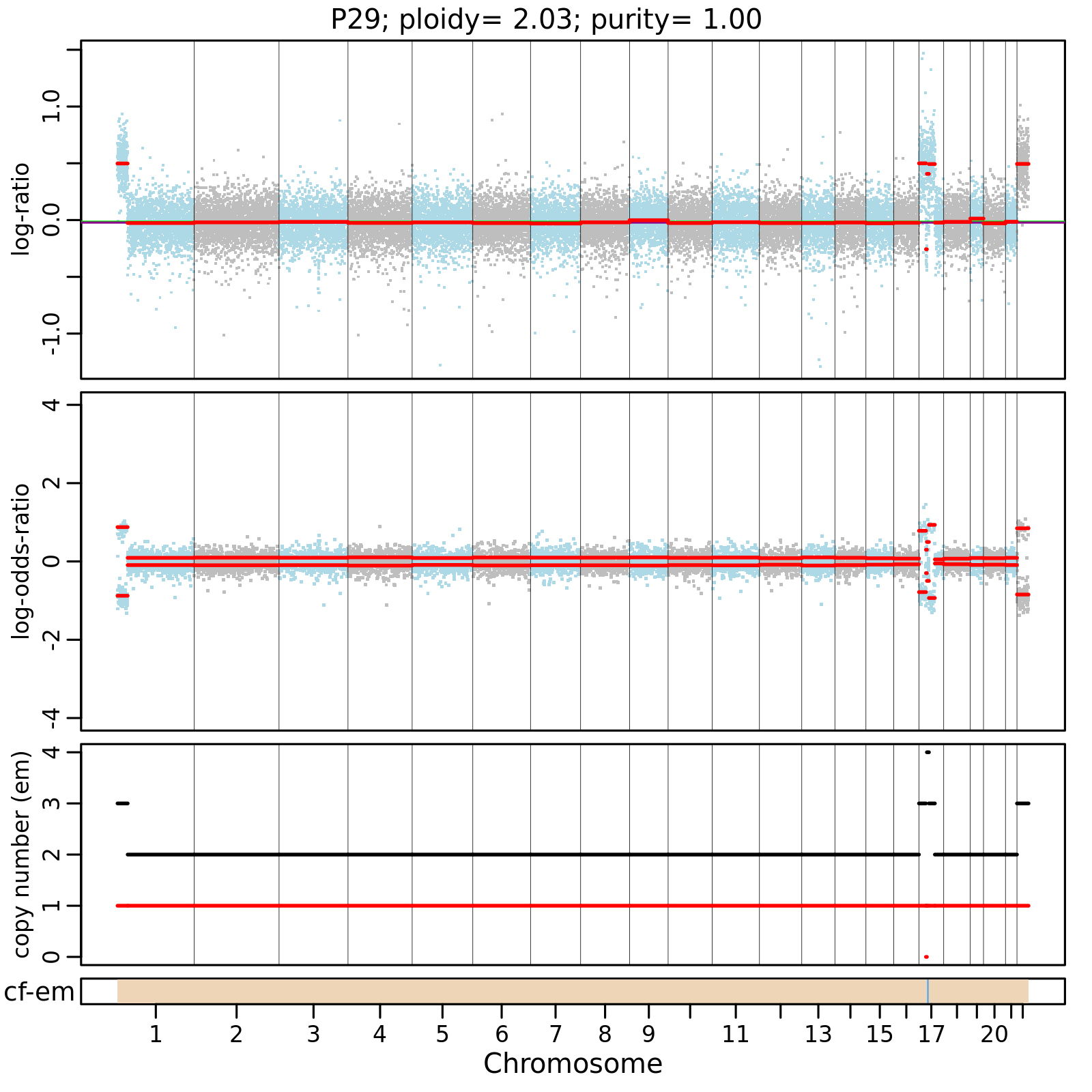

Supplement: Supplementary file 1 [file DataSheet1.ZIP › CNV_plot/P29.cnv.png]
